# Neural Generators Underlying Temporal Envelope Processing Show Altered Responses and Hemispheric Asymmetry Across Age

**DOI:** 10.3389/fnagi.2020.596551

**Published:** 2020-12-04

**Authors:** Ehsan Darestani Farahani, Jan Wouters, Astrid van Wieringen

**Affiliations:** Research Group Experimental Oto-rhino-laryngology (ExpORL), Department of Neurosciences, Katholieke Universiteit Leuven, Leuven, Belgium

**Keywords:** aging, neural generators, auditory temporal processing, ASSR, auditory steady-state response, EEG

## Abstract

Speech understanding problems are highly prevalent in the aging population, even when hearing sensitivity is clinically normal. These difficulties are attributed to changes in central temporal processing with age and can potentially be captured by age-related changes in neural generators. The aim of this study is to investigate age-related changes in a wide range of neural generators during temporal processing in middle-aged and older persons with normal audiometric thresholds. A minimum-norm imaging technique is employed to reconstruct cortical and subcortical neural generators of temporal processing for different acoustic modulations. The results indicate that for relatively slow modulations (<50 Hz), the response strength of neural sources is higher in older adults than in younger ones, while the phase-locking does not change. For faster modulations (80 Hz), both the response strength and the phase-locking of neural sources are reduced in older adults compared to younger ones. These age-related changes in temporal envelope processing of slow and fast acoustic modulations are possibly due to loss of functional inhibition, which is accompanied by aging. Both cortical (primary and non-primary) and subcortical neural generators demonstrate similar age-related changes in response strength and phase-locking. Hemispheric asymmetry is also altered in older adults compared to younger ones. Alterations depend on the modulation frequency and side of stimulation. The current findings at source level could have important implications for the understanding of age-related changes in auditory temporal processing and for developing advanced rehabilitation strategies to address speech understanding difficulties in the aging population.

## Introduction

Speech understanding becomes increasingly challenging with age. Many middle-aged and older people experience difficulties following conversations, especially in noisy environments or when multiple speakers are talking simultaneously. While aging is often accompanied by loss of hearing sensitivity in the high frequencies, these difficulties occur even with normal hearing sensitivity most probably due to age-related changes in central temporal processing (e.g., Presacco et al., 2015, 2019; Du et al., 2016; Goossens et al., 2016; Roque et al., 2019). For e.g., Ostroff et al. (2003) showed alterations in the central processing of sound duration in older adults as revealed by the amplitude of N1 and P2 waves of auditory evoked potentials. Ross et al. (2010) reported a decline in high-gamma oscillations in the central auditory system with progressing age during the processing of a gap in a continuous sound. Moreover, responses to sound envelopes in the central auditory system also alter with progressing age (Walton et al., 2002; Presacco et al., 2015, 2019; Goossens et al., 2016; Parthasarathy et al., 2019). These altered responses occur in older listeners with clinically normal audiometric thresholds (Presacco et al., 2015; Goossens et al., 2016), as well as in persons with peripheral hearing loss (Millman et al., 2017; Goossens et al., 2019). In order to gain a better understanding of age-related changes in central temporal processing, particularly envelope encoding, it is of great interest to disentangling the effect of aging and hearing loss via recruiting older participants who are relatively free of hearing loss.

Speech signals contain various important modulations, of which the temporal envelope of speech (slow fluctuations 2–50 Hz) is essential for accurate speech understanding (Drullman et al., 1994; Shannon et al., 1995; Stone et al., 2010; Peelle and Davis, 2012) and transmits both prosodic and linguistic information (Rosen, 1992). Temporal processing is mediated by synchronized (phased-locked) neural activity (Luo and Poeppel, 2007; Cogan and Poeppel, 2011) and can be investigated electrophysiologically through the auditory steady-state responses (ASSRs), auditory evoked responses that reflect neural synchrony. The electroencephalogram (EEG) is highly suitable to study auditory temporal processing mainly because of the excellent temporal resolution and the rich information about the dynamics of responses (Picton, 2013; He et al., 2018).

Previous studies suggest that age-related changes in neural envelope processing may, in part, underlie impaired speech perception of older persons, with no change or enhanced ASSR amplitudes for slow modulations (<50 Hz) and decreased responses for fast modulations (Leigh-Paffenroth and Fowler, 2006; Tlumak et al., 2015; Goossens et al., 2016). What remains unclear is how age affects the different subcortical and cortical neural generators in response to slow and fast modulations and whether potential changes are discernible at midlife. ASSRs can have cortical and subcortical sources along the auditory pathway (Steinmann and Gutschalk, 2011; Overath et al., 2012; Ross et al., 2020). In response to low modulation frequencies, more activity in the cortical sources [auditory cortex (AC)] than subcortical sources is observed, while for high modulation frequencies, the subcortical sources are more active (Giraud et al., 2000; Liégeois-Chauvel et al., 2004). At the cortical level, not only primary sources in the AC are involved in the generation of ASSRs but also sources outside of the AC, designated as non-primary ones (Farahani et al., 2017, 2019, 2020). However, due to the volume conduction of the brain tissue, a weighted average of activity of different neural generators is recorded at sensor-level data. Via brain source analysis, we can estimate the original neural activity of each generator separately. The rich information unraveled via source analysis increases our understanding of the age-related changes at different levels of the auditory pathway. Moreover, source analysis enables us to investigate less active neural generators at different modulation frequencies, i.e., subcortical generators at low modulation frequencies or cortical activity at high modulation frequencies. This is less straightforward through sensor-level analysis. This research is novel and unique, as the age-related changes in temporal envelope processing at different levels of the auditory pathway, separately, have not been reported yet.

Generally, aging is accompanied by decreasing inhibitory neurotransmission across central auditory regions (Caspary et al., 2008, 2013). In turn, the loss of normal inhibition causes age-related changes in the response properties of neurons in the auditory pathway. In the same vein, electrophysiological studies on aging auditory systems found different alterations in the processing of acoustic modulations and explained them in the context of loss of normal inhibition. The older group showed a degraded temporal precision and neural synchronization (phase-locking) in following the rapidly changing speech envelopes or fast modulations (Anderson et al., 2012; Herrmann et al., 2017), not slow modulations (Herrmann et al., 2017). Aging was also associated with enhanced neural responses to simple (e.g., tones and noise bursts) and slow stimuli (Parthasarathy et al., 2010, 2019; Herrmann et al., 2017). Investigating potential changes in neural generators across different age groups is essential to determine the onset and progression of altered temporal processing and subsequently to define appropriate care.

Auditory processing reveals a hemispheric asymmetry for different phonological segments of speech. For example, the syllables modulations rate (around 4 Hz) is assumed to be processed predominantly in the right hemisphere, while the phonemes modulations rate (around 20 Hz) is processed more in the left hemisphere (e.g., Poeppel, 2003). Changes to this hemispheric asymmetry may account for impaired speech perception, as suggested for dyslexia research for example (Hämäläinen et al., 2012). Studying the potential age-related alterations in hemispheric asymmetry of older adults can provide new insights into the aging auditory system. There is some evidence for the association of age with alternations in hemispheric asymmetries for high-level auditory and cognitive processing in adults (Greenwald and Jerger, 2001; Cabeza, 2002; Berlingeri et al., 2013). However, little is known about how age affects hemispheric asymmetry for different neural generators, particularly for those in response to low-level auditory processing.

The aim of the current research is to investigate changes in auditory temporal processing for a wide range of neural sources in young, middle-aged, and older persons with normal audiometric thresholds. While aging is often accompanied by decreasing audiometric thresholds in the high-frequency region (presbycusis), clinically normal audiometric thresholds are an important prerequisite to disentangle the potential effects of age and hearing loss. In line with the animal studies and electrophysiological research discussed previously, we hypothesize (1) that the neural generators of ASSRs in older adults with clinically normal audiometric thresholds will show enhanced response strength compared to younger adults, while phase-locking will not change noteworthy when the modulations are relatively slow (<50 Hz) and (2) that phase-locking of faster modulations (>50 Hz) may be diminished, together with a reduced response strength. Because of known loss of inhibition in middle-aged animals (Caspary et al., 2008, 2013), we expect changes in phase-locking and response strength to appear at midlife. With regard to hemispheric asymmetry in temporal envelope processing, we hypothesize that the source analyses of ASSRs across different age cohorts would reveal some age-related changes. However, these changes may vary depending on the modulation frequency.

Primary and non-primary neural sources of ASSRs are reconstructed using a minimum-norm imaging (MNI) approach (Farahani et al., 2020). For each neural source, the ASSR amplitude and phase coherence (inter-trial phase coherence) are calculated to investigate the response strength and the phase-locking to the stimulus, respectively. This is done for slow and fast acoustic modulations, presented to both the left and right ears to investigate potential altered hemispheric processing (Cabeza, 2002; Ross, 2008).

## Materials and Methods

### Participants

The EEG data were adopted from Goossens et al. (2016). Participants were in three narrow age cohorts including 19 young (20–30 years, nine men), 20 middle-aged (50–60 years, 10 men), and 16 older adults (70–80 years, five men). Compared to the study of Goossens et al. (2016), two recordings were added to the older cohort.

All participants had normal hearing in both ears, with audiometric thresholds within the clinically normal limits [≤25 dB HL] at all octave frequencies from 125 Hz up to and including 4 kHz. However, young participants had statistically better audiometric thresholds compared to middle-aged and older adults (Goossens et al., 2016). Goossens et al. (2016), using partial correlation analysis, showed that these differences in peripheral hearing do not mediate the observed alterations in temporal envelope processing across the three age groups.

All participants were also screened for mild cognitive impairment by means of the Montreal Cognitive Assessment Task (Nasreddine et al., 2005) and the cutoff score of 26 out of 30. The stringent cutoff score provides excellent sensitivity for detecting mild cognitive impairments (Nasreddine et al., 2005). This screening ensured that all participants had cognitive capacities within the normal range. All participants were Dutch native speakers and right-handed as assessed by the Edinburgh Handedness Inventory (Oldfield, 1971), and none of them has a medical history of brain injury, neurological disorders, or tinnitus.

The participants recruited in the current study, older participants in particular, were selected to have exceptionally good hearing and cognition. These strict selection criteria were important to research differences in auditory neural processing across age in the absence of cognitive and hearing difficulties. In the older cohort, only 16 out of 227 hearing-screened candidates (7%) satisfied all criteria and were included for EEG testing. This low rate was predictable, as only 10% of men and 50% of women older than 70 years old have thresholds ≤25 dB HL up to and including 4 kHz (International Organization for Standardization, 2000).

### Stimuli

To generate stimuli, white noise (bandwidth of 1 octave, centered at 1 kHz) was 100% sinusoidally amplitude modulated at 3.91, 19.53, 40.04, and 80.08 Hz. These values were chosen to ensure that there is an integer number of cycles in an epoch of 1.024 s (John and Picton, 2000). The modulations around 4 and 20 Hz were presented as a model of the rate of occurrence of syllables and phonemes, respectively. Relatively high modulations at 40 and 80 Hz were also selected because it was shown that these modulations can activate more subcortical neural generators than cortical ones (Giraud et al., 2000; Herdman et al., 2002).

The stimuli were presented at 70 dB SPL via ER-3A insert phones. Every stimulus type was presented one time to the left ear and another time to the right ear, each time for 300 s continuously. The order of stimulus presentation was randomized between participants.

### Experimental Procedure

The experiment protocol was designed to ensure passive listening to amplitude-modulated (AM) stimuli during a wakeful state. During acoustic stimulation, participants were lying on a bed and watching a muted movie with subtitles. The movie was displayed on a 21-inch LCD monitor with 60 Hz vertical refresh rate. We asked participants to lie on a bed in order to prevent possible movement caused by fatigue, especially with older participants. A large-size and very soft pillow was used to support the neck and a big area of the head. Thus, no focal pressure on the occipital electrodes and interference with recording were expected. In order to prevent movements and muscle tensions, the participants were encouraged to lie quietly and relaxed during auditory stimulation. Moreover, the electrode offset values were continuously monitored during the measurements and kept below ±30 mV to be sure about the proper electrode contact. The procedure was performed in a double-walled soundproof booth with a Faraday cage.

The EEG signals were recorded using the BioSemi ActiveTwo system including 64 active pin-type electrodes that were fixed in the head cap based on the 10–10 electrode system. The electrodes CMS and DRL act as the common electrode and current return path, respectively. The EEG signals were amplified and recorded at a sampling rate of 8,192 Hz with a gain of 32.25 nV/bit. The system uses a built-in low-pass filter with a cutoff frequency of 1,638 Hz.

### EEG Source Analysis

The brain sources of ASSRs were reconstructed using a variety of MNI, which was adapted for ASSR source analysis (Farahani et al., 2020). An overview of this approach is given below [for more details, see Farahani et al. (2020)].

#### Pre-processing

EEG data were preprocessed in MATLAB R2016b (MathWorks). The continuous EEG data were filtered by a zero phase high-pass filter with a cutoff frequency of 2 Hz (Butterworth, 12 dB/octave) to attenuate the low-frequency distortions and drift of the amplifier. The filtered data were segmented into epochs of 1.024 s. Subsequently, early noise reduction was performed in three steps, as follows:

1. Channel rejection: for each of the 64 EEG channels, the mean of the maximum absolute amplitude of all epochs was obtained as an index of “maximum amplitude.” The channels with maximum amplitude index more than 100 μV were rejected.
2. Recording rejection: a recording was excluded from further analyses if it had more than five (out of 64) rejected channels. On average, 1.6 (standard deviation of 1) recordings were excluded across the three age cohorts, four modulation frequencies, and two sides of stimulations.
3. Epoch rejection: the highest peak-to-peak (PtoP) amplitude of the signals in the remaining channels was calculated for each epoch separately and considered as an index of PtoP. The epochs were sorted based on PtoP amplitude, and 10% of them with the highest PtoP amplitudes were rejected.

Afterward, the EEG data were re-referenced to a common average over all remaining channels and epochs. Independent component analysis (ICA) based on the Infomax algorithm as implemented in the Fieldtrip toolbox (Oostenveld et al., 2011) was applied to the re-referenced data to attenuate artifacts caused by eye blinks, eye movements, and heartbeats. The noisy components were recognized by visual inspection and removed from subsequent reconstruction. Subsequently, the removed channels were interpolated using the spherical spline method (Perrin et al., 1989) implemented in the Fieldtrip toolbox (Oostenveld et al., 2011). The order of interpolation and the regularization parameter were set to 3 and 10^−8^, respectively, as is suggested by Kang et al. (2015), to minimize distortions in temporal features of interpolated channels. Lastly, the remaining artifactual epochs not accounted for by ICA were identified and removed using a threshold level of 70 μV for maximum absolute amplitude of each epoch. To have an equal number of epochs across participants, only the first 192 artifact-free epochs (six sweeps of 32 epochs) of each participant were preserved for subsequent analyses. If <192 epochs could be retained, the threshold level was gradually increased (in steps of 5 μV and up to maximally 110 μV) until at least 192 artifact-free epochs were identified.

#### Sources Reconstruction: Developing ASSR Map

##### Reconstruction Source Map of EEG in Time Domain

The source distribution map was estimated using dynamic statistical parametric mapping (dSPM; Dale et al., 2000) implemented in the Brainstorm application (Tadel et al., 2011, 2019). In dSPM, the standard minimum-norm solution is normalized with the estimated noise at each source (Lin et al., 2006). This noise normalization attenuates the bias toward superficial sources, which is the inherent property of the standard minimum norm solution (Lin et al., 2006; Hauk et al., 2011).

##### Noise Covariance Matrix

The noise covariance matrix required for noise normalization was obtained from the silence EEG data, i.e., the EEG recording in the absence of auditory stimulation. The silence data of participants were band-pass filtered (zero-phase with a bandwidth of 4 Hz and modulation frequency as center frequency) and concatenated to calculate the covariance matrix.

##### Mixed Head Model

In order to reconstruct both cortical and subcortical sources, a mixed head model consisting of cortical and subcortical regions was generated. This head model was obtained from the template anatomy ICBM152 (Fonov et al., 2011) and the default channel location file in Brainstorm using the boundary element method (BEM), as implemented in OpenMEEG (Gramfort et al., 2010).

##### Data Averaging for Group-Wise Analyses

When a head model is generated based on template anatomy, group-wise source analysis can lead to a higher localization accuracy of neural generators than individual-level analyses (Farahani et al., 2019). In the current study, the artifact-free epochs of each participant were divided into six sweeps of 32 concatenated epochs and averaged across participants to perform a group-wise analysis. The obtained grand-averaged sweep was used for dSPM.

##### Regularization Parameter

The regularization parameter (λ^2^) required for dSPM was obtained as:

(1) $$\begin{array}{l} \lambda^{2}=\frac{1}{SNR_{scalp}^{2}\;} \end{array}$$

where *SNR*_*scalp*_ is the signal-to-noise ratio (SNR) (based on the amplitude) of the whitened EEG data (Bradley et al., 2016; Hincapié et al., 2016; Ghumare et al., 2018). The whitening operator was obtained from Brainstorm. For each EEG channel, the ASSR strength (amplitude at the modulation frequency) was obtained from the fast Fourier transform (FFT). The maximum response amplitude across channels was assigned to the signal of interest (Farahani et al., 2020). The background noise of each channel was estimated based on the average of 30 neighboring frequency bins on the left and the right side of the response frequency bin. The median of the background noise across channels was assigned to the noise level (Farahani et al., 2020).

##### Developing the ASSR Map

The reconstructed source map by dSPM was transformed to the frequency domain by applying FFT to the time-series of each dipole (Farahani et al., 2020). Subsequently, the SNR of the ASSR for each dipole was calculated according to Equation 2.

(2) $$\begin{array}{l} SNR\left(dB\right)=10log_{10}\left(\frac{P_{S+N}}{P_{N}}\right) \end{array}$$

where P_S+N_ is the spectral power at the modulation frequency, which shows the power of the response plus neural background noise. P_N_ refers to the power of the neural background noise, which was estimated on the basis of the mean power of 30 neighboring frequency bins (corresponding to 0.92 Hz) on each side of the modulation frequency bin.

For each dipole, the one sample *F*-test with the SNR (i.e., P_S+N_ / P_N_) as F ratio statistic was used to recognize the dipoles with significant ASSRs (Dobie and Wilson, 1996; John and Picton, 2000; Picton et al., 2005). Results were corrected for multiple comparisons using the false discovery rate (FDR) method (Benjamini and Hochberg, 1995). Subsequently, an ASSR map was generated, which illustrates ASSR amplitudes for dipoles with significant responses and zero for the dipoles with no significant responses. The ASSR amplitude was calculated using Equation 3. For subcortical regions, the ASSR amplitude of each point was calculated based on the norm of the vectorial sum of the three ASSR amplitudes across x, y, z at that point (Equation 4).

(3) $$\begin{array}{l} ASSRamp=\sqrt{P_{S+N}}-\sqrt{P_{N}} \end{array}$$

(4) $$\begin{array}{l} SubcorticalASSRamp=\sqrt{ASSRamp_{x}^{2}+ASSRamp_{y}^{2}+ASSRamp_{z}^{2}} \end{array}$$

**Figure 1:**
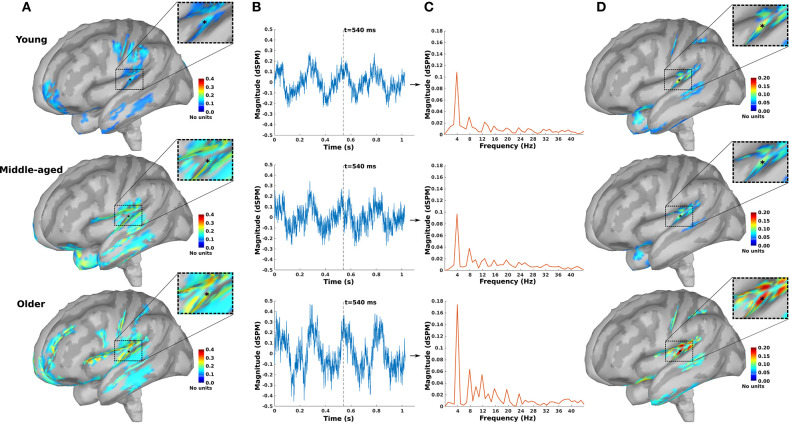
The auditory steady-state response (ASSR) map in response to 4 Hz amplitude-modulated (AM) stimuli presented to the right ear across age. **(A)** Reconstructed source map at 540 ms using dynamic statistical parametric mapping (dSPM) and enlarged view of a sample dipole located in the auditory cortex [−35, −28, 16, xyz in Montreal Neurological Institute (MNI) coordinates]. The map illustrates the absolute values of activity, and the color bar indicates the magnitude of activity (no unit because of normalization, which is performed within the dSPM algorithm). **(B)** Time-series of the sample dipole (original values with length of one epoch) for the three age cohorts. The vertical dashed line indicates the time point of 540 ms. **(C)** The frequency spectrum of the sample dipole for the three age cohorts. **(D)** The generated ASSR map for the three age cohorts. The color bar indicates the ASSR amplitude.

As an example, the source maps at 540 ms in response to 4 Hz AM stimuli presented to the right ear for young, middle-aged, and older cohorts are shown in Figure 1A. The time-series of all dipoles were transformed into the frequency domain to calculate the ASSR amplitudes (based on Equations 3, 4), and the outcomes were used to develop the ASSR map. The time-series of a sample dipole located in the AC and its frequency response for the three age cohorts are shown in Figures 1B,C, respectively. In Figure 1D, the generated ASSR map of older participants shows a higher ASSR amplitude in the AC than those of young and middle-aged participants.

In addition to the ASSR map, the SNR map was also generated. This map illustrates the SNR (in dB, Equation 2) for the dipoles with significant ASSRs and zero for the dipoles with no significant responses.

#### Defining Regions of Interest

**Figure 2:**
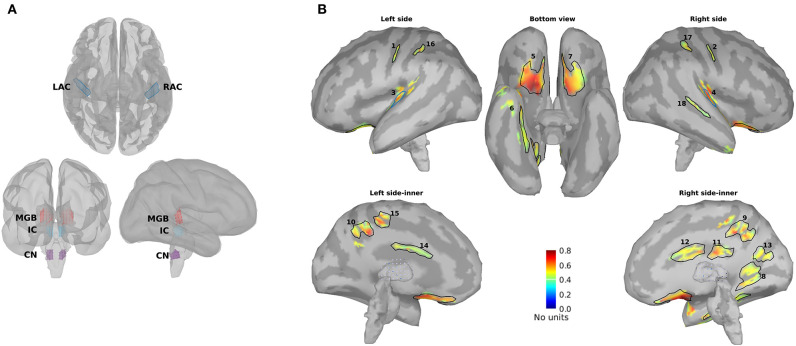
Primary and non-primary regions of interest (ROIs). **(A)** The primary ROIs are located bilaterally in the auditory cortex (LAC, RAC), the medial geniculate body (LMGB, RMGB), inferior colliculus (LIC, RIC), and cochlear nucleus (LCN, RCN). **(B)** The non-primary ROIs were based on the averaged normalized signal-to-noise ratio (SNR) maps of all experimental conditions [young, middle aged, and older cohort, 4, 20, 40, and 80 Hz amplitude-modulated (AM) stimuli presented to the left and the right ears] and the obtained ROIs. The anatomical labels of the primary and the non-primary ROIs are listed in Table 1.

For further analysis and comparison of the ASSR maps, we need to define regions of interest (ROIs). Eight ROIs were defined along the primary auditory pathway on the basis of the anatomical locations of the cochlear nucleus (CN), the inferior colliculus (IC), the medial geniculate body (MGB), and the AC bilaterally (Figure 2A). It has been shown that these regions play a key role in generating ASSRs (Langers et al., 2005; Steinmann and Gutschalk, 2011; Overath et al., 2012; Coffey et al., 2016). At the cortical level, the ROIs of the AC were defined bilaterally in the Heschl's gyrus (left AC: 5.49 cm^2^; right AC: 5.58 cm^2^) with reference to the transverse temporal gyrus in the Desikan–Killiany atlas implemented in Brainstorm (Desikan et al., 2006; Tadel et al., 2011). The subcortical ROIs were defined bilaterally in the CN (estimated with reference to the medullary pontine junction; left CN: 0.49 cm^3^; right CN: 0.47 cm^3^), IC (recognized with reference to the thalamus; left IC: 0.50 cm^3^; right IC: 0.55 cm^3^), and in the posterior thalamus (roughly the posterior third of the thalamus; left MGB: 1.24 cm^3^; right MGB: 1.45 cm^3^) (Coffey et al., 2016; Farahani et al., 2020).

In addition to the primary ROIs, some other ROIs were defined according to the average SNR maps across all three age cohorts. These ROIs were termed non-primary ROIs because they were located outside of the AC (Farahani et al., 2020). Firstly, the SNR index [with a range of (0,1)] of each dipole (s) was calculated according to Equation 5 to have the same dynamic ranges of SNR across different experimental conditions (three age cohorts, four modulation frequencies, and two sides of stimulations). *SNRmax* and *SNRmin* in Equation 5 show, respectively, the maximum and the minimum value of SNR (in dB) on that specific map.

(5) $$\begin{array}{l} SNRindex\left(s\right)=\frac{SNR\left(s\right)-SNRmin}{SNRmax-SNRmin} \end{array}$$

Afterward, the new maps based on the SNR index were generated and averaged across different experimental conditions. Lastly, the regions of the grand-averaged map with an SNR index of more than 50% of the range were selected as ROIs (Figure 2B). The respective anatomical labels of the primary and the non-primary ROIs are listed in Table 1.

**Table 1:** Anatomical label of primary and non-primary regions of interest (ROIs).

**Primary ROIs**	**Non-primary ROIs**	
**Cortical:** #3 Left auditory cortex (LAC) #4 Right auditory cortex (RAC) **Subcortical:** #19 Left medial geniculate body (LMGB) #20 Right medial geniculate body (RMGB) #21 Left inferior colliculus (LIC) #22 Right inferior colliculus (RIC) #23 Left cochlear nucleus (LCN) #24 Right cochlear nucleus (RCN)	#1 Left precentral gyrus (LPrC) #2 Right precentral gyrus (RPrC) #5 Right orbitofrontal (ROF) #6 Right parahypocampal (RPHC) #7 Left orbitofrontal (LOF) #8 Right occipital (ROcc) #9 Right superior parietal (RSP) #10 Left superior parietal (LSP)	#11 Right posterior cingulate gyrus (RPCG) #12 Right anterior cingulate gyrus (RACG) #13 Right parieto-occipital (RPO) #14 Left cingulate gyrus (LCG) #15 Left paracentral gyrus (LPG) #16 Left postcentral gyrus (LPoC) #17 Right postcentral gyrus (RPoC) #18 Right middle temporal gyrus (RMTG)

#### Time-Series of ROIs and ASSR Amplitude

In order to compare different experimental conditions, we need to extract a time-series for each ROI. To this end, a representative dipole inside each ROI was identified using the algorithm suggested by Farahani et al. (2020). In this algorithm, first, a response patch with the highest mean ASSR amplitude was selected for each ROI. Subsequently, among the dipoles of the selected response patch, a representative dipole with the most similar ASSR (regarding amplitude and phase of the response) to the mean ASSR of the patch was chosen. The ASSR amplitudes of the representative dipoles were used for further analyses.

In the subcortical ROIs, the ASSR amplitudes of dipoles were very close to their neighboring dipoles. Therefore, to decrease the computational load, the dipole with the highest ASSR amplitude was selected as representative dipole. Subsequently, three reconstructed time-series (x-, y-, and z-components) accounting for the representative dipoles were extracted for further analysis. The ASSR amplitudes of the representative dipoles in the subcortical level were obtained based on the Euclidean norm of the amplitudes of x-, y-, and z-components.

### Phase Coherence

Phase coherence (or inter-trial phase coherence) shows the similarity in the phase of ASSRs across epochs (Picton et al., 2001; Luo and Poeppel, 2007). It also reflects the phase-locking capability of a neural source to the acoustic stimulus and varies between 0 and 1 (Koerner and Zhang, 2015; Farahani et al., 2019). For each ROI, the phase coherence was calculated based on the time-series of the representative dipoles of that ROI. The extracted time-series (192 epochs) were divided into 64 groups of three epochs. The ASSR phase of each epoch was obtained from the complex form of response in the frequency domain. Phase coherence was calculated according to Equation 6, where θ_i_ refers to the phase of group *i (i* = *1, 2, …, 64)* obtained from the complex responses averaged across the three epochs (Picton et al., 2001).

(6) $$\begin{array}{l} Phasecoherence=\frac{1}{N}\sqrt{{\left(\overset{N}{\underset{i=1}{\sum }}\cos\theta_{i}\right)}^{2}+{\left(\overset{N}{\underset{i=1}{\sum }}\sin\theta_{i}\right)}^{2}} \end{array}$$

For each subcortical ROI, we have three reconstructed time-series (x-, y-, and z-components), while we need to have only one time-series per ROI. To this end, we estimated the optimal dipole direction accounting for most of the variance of the ASSR activity by means of singular value decomposition (SVD) (Rueda-Delgado et al., 2017). First, the three time-series were filtered using a Butterworth zero phase band-pass filter with a bandwidth of 4 Hz and modulation frequency as center frequency. Then, SVD was applied to the filtered data to find the optimal direction. The projection of the three components on the optimal direction was used for calculating the phase coherence.

### Hemispheric Lateralization

Functional hemispheric asymmetry was determined using the laterality index (LI). The LI is a normalized index with the range of [-1,1], where positive values show lateralization to the right hemisphere. LI was calculated for the bilateral sources as:

(7) $$\begin{array}{l} LI=\frac{ASSRamp_{R}-ASSRamp_{L}}{ASSRamp_{R}+ASSRamp_{L}} \end{array}$$

where *ASSRamp*_*R*_ and *ASSRamp*_*L*_ indicate the ASSR amplitude (based on all participants, Equations 3, 4) of the source located in the right and left hemispheres, respectively. In order to avoid lateralization errors, the LI was only calculated when both sources had a significant ASSR.

The variation of LI was estimated using the jackknife method. In this method, each jackknife resample of LI was calculated using the resamples of *ASSRamp*_*R*_ and *ASSRamp*_*L*_.

### Statistical Analyses

The standard deviations of the ASSR amplitudes, phase coherence, and LI were estimated separately using the jackknife resampling method (Efron and Stein, 1981). For each resample of participants, the main dSPM imaging kernel was applied to the averaged EEG data of that resample. The mean of each measure (ASSR amplitudes, phase coherence, and LI) was obtained based on the entire group of participants without resampling. All statistical analyses were based on the mean, estimated standard deviation, and the number of participants, rather than on individual scores (Cohen, 2002; Nagy, 2013) using custom scripts in MATLAB R2016b (MathWorks).

To investigate the overall effect of age on ASSR amplitude, a factorial mixed analysis of variance (FM-ANOVA) with side of stimulation (two levels: left and right) and ROIs (24 levels: eight primary and 16 non-primary) as within-subject variables was carried out for 4, 20, 40, and 80 Hz ASSRs, separately. *Post hoc* testing was performed using a two-sample *t*-test with Bonferroni correction. In *post hoc* testing with ROIs as within-subject variable, the statistical tests often demonstrated a significant difference due to the large sample size. So, the effect sizes, Cohen's d, were also reported to provide a measure of significance independent of sample size. Moreover, reporting the effect sizes was strongly advised (Sullivan and Feinn, 2012). Cohen's d was used as a measure of effect size. Age-related changes of ASSR amplitude were also investigated for four categories of ROIs (Table 1), namely, primary, cortical, subcortical, and non-primary ROIs. Similar statistical analyses were performed for phase coherence.

For hemispheric lateralization, a series of one-sample *t*-tests were performed per side of stimulation and per modulation frequency to investigate which ROIs exhibited an LI significantly different from zero. The results were corrected for multiple comparisons using the FDR method (Benjamini and Hochberg, 1995). A significant positive (negative) LI indicated a right (left) hemisphere laterality, and a non-significant LI showed a symmetrical response pattern. A possible effect of age was investigated using FM-ANOVA plus *post hoc* testing per modulation frequency and per side of stimulation.

## Results

### Effect of Age on the Response Strength of the Neural Sources

**Figure 3:**
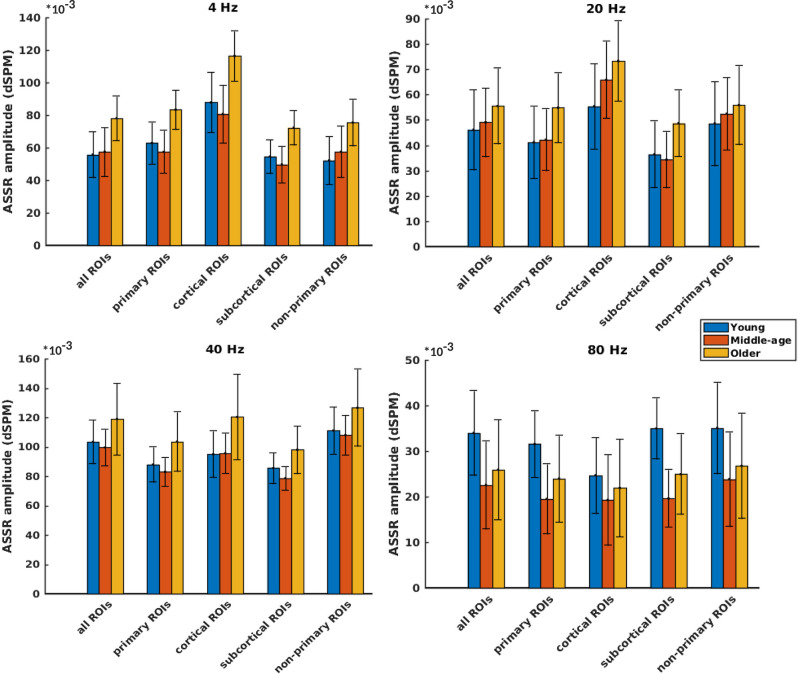
Auditory steady-state response (ASSR) amplitudes of different categories of sources (Table 1) regardless of side of stimulation across age and across modulation frequency. The bars indicate the weighted average of amplitudes (number of subjects as weights), and error bars represent the pooled standard deviations (Cohen, 1988). Forty-eight of 60 comparisons showed significant difference (Table 2).

Figure 3 illustrates the mean response strengths for “all ROIs” as well as for different categories (primary, cortical, subcortical, and non-primary) for each of the four different modulation frequencies. A significant main effect of age was observed for all ROIs and also different categories of ROIs in all acoustic conditions. *Post hoc* testing analyses revealed significantly larger response strengths for the older compared to the young and middle-aged participants in all categories for the **4 Hz** modulation frequency. The effect size of the mean difference (Cohen's d) was very similar across different categories, specifically when comparing young and older participants. The results of *post hoc* testing are summarized in Table 2.

**Table 2:** The results of *post hoc* comparisons of auditory steady-state response (ASSR) amplitude and phase coherence across age in different categories of sources [all regions of interest (ROIs), primary, cortical, subcortical, and non-primary].

		**ASSR amplitude**			**Phase coherence**		
	**Category**	**Young, middle-aged**	**Young, older**	**Middle-aged, older**	**Young, middle-aged**	**Young, older**	**Middle-aged, older**
4 Hz	All ROIs	*d* = −0.1	*d* = −1.6	*d* = −1.4	*d* = −0.2	*d* = −0.2	*d* < −0.1
		TD	*p* < 0.001	*p* < 0.001	TD	TD	TD
	Primary	*d* = 0.4	*d* = −1.6	*d* = −2	*d* = 0.1	*d* = 0.2	*d* = −0.2
		*p* < 0.001	*p* < 0.001	*p* < 0.001	TD	TD	TD
	Cortical	*d* = 0.3	*d* = −1.6	*d* = −2.1	*d* = 0.2	*d* = −0.6	*d* = −0.8
		*p* < 0.05	*p* < 0.001	*p* < 0.001	TD	*p* < 0.001	*p* < 0.001
	Subcortical	*d* = 0.4	*d* = −1.6	*d* = −2	*d* = 0.1	*d* = 0.1	*d* = 0.1
		*p* < 0.001	*p* < 0.001	*p* < 0.001	TD	TD	TD
	Non-primary	*d* = −0.3	*d* = −1.6	*d* = −1.1	*d* = −0.3	*d* = −0.4	*d* < −0.1
		*p* < 0.001	*p* < 0.001	*p* < 0.001	*p* < 0.001	*p* < 0.001	TD
20 Hz	All ROIs	*d* = −0.2	*d* = −0.6	*d* = −0.4	*d* = −0.4	*d* = −0.2	*d* = 0.1
		TD	*p* < 0.001	*p* < 0.001	*p* < 0.001	TD	TD
	Primary	*d* = −0.1	*d* = −1.0	*d* = −1.0	*d* = −0.3	*d* = −0.6	*d* = −0.2
		TD	*p* < 0.001	*p* < 0.001	*p* < 0.001	*p* < 0.001	TD
	Cortical	*d* = −0.6	*d* = −1.0	*d* = −0.4	*d* = −0.5	*d* < −0.1	*d* = 0.4
		*p* < 0.001	*p* < 0.001	*p* < 0.01	*p* < 0.01	TD	*p* < 0.01
	Subcortical	*d* = 0.1	*d* = −0.9	*d* = −1.1	*d* = −0.2	*d* = −0.7	*d* = −0.5
		TD	*p* < 0.001	*p* < 0.001	TD	*p* < 0.001	*p* < 0.001
	Non-primary	*d* = −0.2	*d* = −0.4	*d* = −0.2	*d* = −0.4	*d* < −0.1	*d* = 0.4
		TD	*p* < 0.001	TD	*p* < 0.001	TD	*p* < 0.001
40 Hz	All ROIs	*d* = 0.2	*d* = −0.7	*d* = −1.0	*d* < 0.1	*d* < −0.1	*d* = −0.1
		TD	*p* < 0.001	*p* < 0.001	TD	TD	TD
	Primary	*d* = 0.4	*d* = −0.9	*d* = −1.3	*d* = 0.2	*d* < 0.1	*d* = −0.1
		*p* < 0.001	*p* < 0.001	*p* < 0.001	TD	TD	TD
	Cortical	*d* = 0.0	*d* = −1.1	*d* = −1.1	*d* = 0.2	*d* = −0.4	*d* = −0.7
		TD	*p* < 0.001	*p* < 0.001	TD	*p* < 0.05	*p* < 0.001
	Subcortical	*d* = 0.7	*d* = −0.9	*d* = −1.5	*d* = 0.2	*d* = 0.2	*d* = 0.1
		*p* < 0.001	*p* < 0.001	*p* < 0.001	TD	TD	TD
	Non-primary	*d* = 0.2	*d* = −0.7	*d* = −0.9	*d* < −0.1	*d* = −0.1	*d* < −0.1
		TD	*p* < 0.001	*p* < 0.001	TD	TD	TD
80 Hz	All ROIs	*d* = 1.2	*d* = 0.8	*d* = −0.3	*d* = 0.7	*d* = 0.9	*d* = 0.1
		*p* < 0.001	*p* < 0.001	*p* < 0.001	*p* < 0.001	*p* < 0.001	TD
	Primary	*d* = 1.6	*d* = 0.9	*d* = −0.5	*d* = 0.4	*d* = 1	*d* = 0.5
		*p* < 0.001	*p* < 0.001	*p* < 0.001	*p* < 0.001	*p* < 0.001	*p* < 0.001
	Cortical	*d* = 0.5	*d* = 0.2	*d* = −0.2	*d* < 0.1	*d* = 0.4	*d* = 0.2
		*p* < 0.001	TD	TD	TD	*p* < 0.05	TD
	Subcortical	*d* = 2.3	*d* = 1.2	*d* = −0.7	*d* = 0.6	*d* = 1.3	*d* = 0.5
		*p* < 0.001	*p* < 0.001	*p* < 0.001	*p* < 0.001	*p* < 0.001	*p* < 0.001
	Non-primary	*d* = 1.1	*d* = 0.8	*d* = −0.2	*d* = 0.8	*d* = 0.9	*d* < −0.1
		*p* < 0.001	*p* < 0.001	TD	*p* < 0.001	*p* < 0.001	TD

*The tests were conducted per modulation frequency. Cohen's d and p-value were reported for different pairs of age cohorts and different modulation frequencies. The trivial difference is indicated by TD and refers to when Cohen's d suggested a small (d ≤0.2) effect size of mean differences (Sullivan and Feinn, 2012)*.

For 20 Hz ASSRs, the difference between young and middle-aged was trivial (effect size *d* ≤ 0.2) for most of the categories (except for the cortical ones), while the difference between young and older participants was significant with similar effect sizes across different categories.

For 40 Hz AM stimuli, *post hoc* testing revealed a significantly larger amplitude in the older compared to the young and middle-aged participants with similar effect sizes across different categories.

For 80 Hz modulation frequency, *post hoc* testing revealed a significantly larger amplitude with the young participants compared to the middle-aged and older participants in most categories, except for cortical ones. The effect size of the mean difference (Cohen's d) for the subcortical category was larger than that for other categories.

Detailed information about the ASSR amplitude of different ROIs across age for the left and right sides of stimulation is illustrated in Supplementary Figures 1, 2, respectively. For each modulation frequency, the statistical test for overall effect of age (considering all ROIs) on the response strength showed significant interactions between age group and side of stimulation and also between age group and ROIs. *Post hoc* testing was performed per side of stimulation and per ROI. The results of *post hoc* comparisons are summarized in Supplementary Table 1. These results were corrected for multiple comparisons using the FDR method (Benjamini and Hochberg, 1995).

In brief, age-related changes in response strength were observed in different categories of ROIs and in all acoustic conditions. For 4, 20, and 40 Hz acoustic modulations, a significantly larger response strength in the older compared to the young and middle-aged participants was found, while at 80 Hz, a significantly smaller response strength in older and middle-aged compared to young participants was detected.

### Effect of Age on the Phase Coherence of the Neural Sources

**Figure 4:**
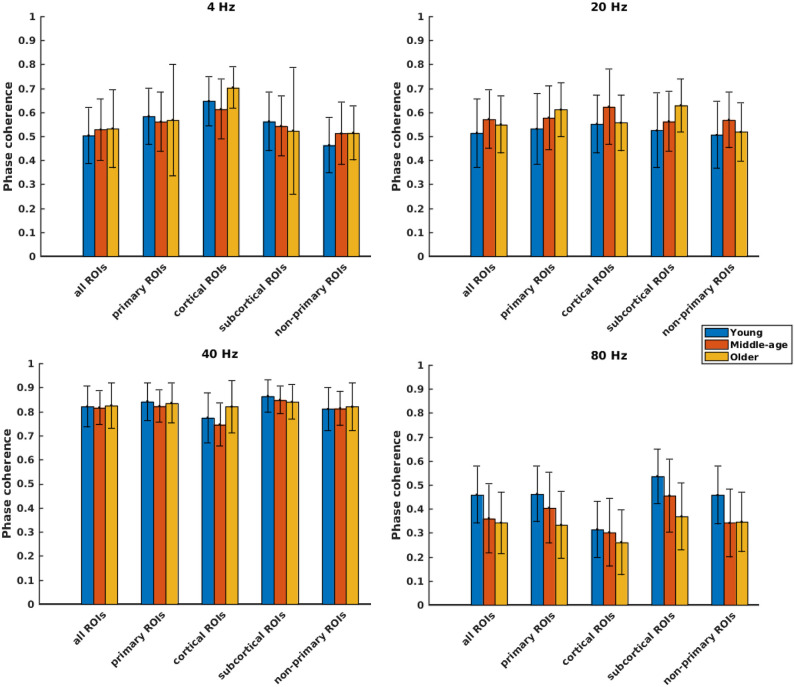
Phase coherence of different categories of sources (Table 1), regardless of side of stimulation across age and across modulation frequency. The bars indicate the weighted average of phase coherence (number of subjects as weights), and error bars represent the pooled standard deviations (Cohen, 1988). Twenty-six of 60 comparisons showed a significant difference (Table 2).

Phase coherence was computed to investigate whether phase-locking differs for the different age cohorts. Figure 4 illustrates the mean phase coherence for “all ROIs” as well as for the four main categories (primary, cortical, subcortical, and non-primary) for each of the four different modulation frequencies. A significant main effect of age was observed for all ROIs and also different categories of ROIs in all acoustic conditions, except for primary and subcortical ROIs at 4 Hz and non-primary ones at 40 Hz. For 4 Hz modulation frequency, *post hoc* testing showed a significantly larger phase coherence in the older compared to the young and middle-aged cohorts in the non-primary and the cortical categories. However, the effect sizes of mean differences (Cohen's d) in these comparisons were small or medium (*d* ≤ 0.5), except for the cortical category. The results of *post hoc* testing are summarized in Table 2.

For 20 Hz ASSR, *post hoc* testing revealed significant effects of age in some categories. However, in most of the comparisons, Cohen's d suggested a small (*d* ≤ 0.2) or medium (*d* ≤ 0.5) effect size of mean differences (Sawilowsky, 2009; Sullivan and Feinn, 2012).

For 40 Hz ASSR, the differences between age groups were trivial (small effect size, *d* ≤ 0.2) across different categories, except for the cortical category where older participants showed higher phase coherence than young and middle-aged participants.

For 80 Hz modulation frequency, *post hoc* testing revealed a significantly larger phase coherence in the young participants compared to the middle-aged and older participants in almost all categories. The middle-aged participants also showed larger phase coherence than the older ones in the primary and the subcortical categories.

Detailed information about the phase coherence of different ROIs across age for the left and right sides of stimulation is illustrated in Supplementary Figures 3, 4, respectively. For each modulation frequency, the statistical test for overall effect of age (considering all ROIs) on the response strength showed significant interactions between age group and side of stimulation and also between age group and ROIs. *Post hoc* testing was performed per side of stimulation and per ROI. The results of *post hoc* comparisons are summarized in Supplementary Table 2. These results were corrected for multiple comparisons using the FDR method (Benjamini and Hochberg, 1995).

Concisely, age-related changes in phase-locking were not noteworthy for 4, 20, and 40 Hz ASSRs, while for 80 Hz ASSRs, a significantly smaller phase-locking was observed in the middle-aged and older participants compared to the young participants in almost all categories.

### Hemispheric Lateralization

**Figure 5:**
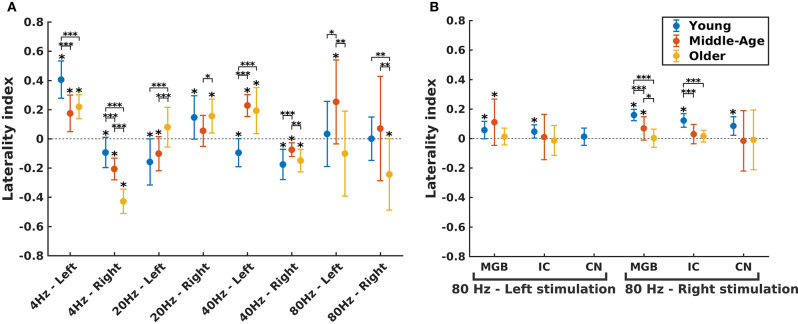
Hemispheric lateralization for different age groups. **(A)** The laterality indexes (LIs) for auditory cortex (AC) across age (indicated by different colors) in different experimental conditions (four modulation frequencies presented to the left or right ear). **(B)** The LIs for the subcortical sources [the medial geniculate body (MGB), the inferior colliculus (IC), the cochlear nucleus (CN)] across age (indicated by different colors) in response to 80 Hz amplitude-modulated (AM) stimuli presented to the left or right ear. LI for CN (left stimulation) was not calculated because of non-significant auditory steady-state response (ASSR) amplitude in one side. The LIs were obtained based on the 17.7 (±0.4), 17.6 (±0.7), and 14.9 (±1) participants in the young, middle-aged, and older cohorts. The error bars illustrate the standard deviations estimated using the jackknife method (Efron and Stein, 1981). The significant left/right lateralization was indicated by an asterisk (*) on top of the error bar. Comparisons across age: *p ≤ 0.05; **p ≤ 0.01; ***p ≤ 0.001.

In order to investigate the functional hemispheric asymmetry across age, the ASSR amplitudes of the left and right auditory cortices were used to calculate the LI. Figure 5 illustrates the LIs of the AC for different stimulation conditions (three age groups, four modulation frequencies, and two sides of stimulation) and LIs of subcortical sources for 80 Hz ASSR. The LIs showing a significant asymmetry to the left or right hemisphere were determined using a one-sample *t*-test (the results are summarized in Supplementary Tables 3, 4).

The effect of age on hemispheric asymmetry was investigated per modulation frequency and per side of stimulation. A significant main effect of age was observed for the different stimulation conditions. The results of *post hoc* testing in different pairs of age cohorts are summarized in Table 3. As shown in Figure 5 and Table 3, the patterns of age-related changes were variable across modulation frequency and sometimes across sides of stimulation. For **4 Hz** AM stimuli presented to the left and right ears, the LIs of the older participants were significantly more negative (toward left hemisphere) than those of the younger and middle-aged ones. However, for **20 Hz** AM stimuli, the hemispheric asymmetry was less or similar for the older participants than for the younger ones in the left and right side of stimulation, respectively. For **40 Hz** AM stimuli presented to the left, the LIs of the older and middle-aged participants were significantly more positive than those of the younger ones, while for the right side of stimulation and also **80 Hz** (both sides), the LIs showed no clear trend, although the data yielded differences depending on the side of stimulation and age.

**Table 3:** The results of *post hoc* comparison of laterality index of auditory cortex across age in different stimulation conditions.

**Stimulation condition**		**Young, middle-aged**	**Young, older**	**Middle-aged, older**
4 Hz	Left ear	*d* = 1.8	*d* = 1.6	NS
		*p* < 0.001	*p* < 0.001	
	Right ear	*d* = 1.2	*d* = 3.5	*d* = 2.8
		*p* < 0.001	*p* < 0.001	*p* < 0.001
20 Hz	Left ear	NS	*d* = −1.6	*d* = −1.4
			*p* < 0.001	*p* < 0.001
	Right ear	NS	*d* < −0.1	*d* = −0.9
			TD	*p* < 0.05
40 Hz	Left ear	*d* = −3.7	*d* = −2.2	*d* = 0.2
		*p* < 0.001	*p* < 0.001	TD
	Right ear	*d* = −1.2	*d* = −0.2	*d* = 1.2
		*p* < 0.001	TD	*p* < 0.01
80 Hz	Left ear	*d* = −0.8	*d* = 0.5	*d* = 1.2
		*p* < 0.05	NS	*p* < 0.01
	Right ear	*d* = −0.2	*d* = 1.2	*d* = 1.0
		TD	*p* < 0.01	*p* < 0.01
	Right ear (MGB)	*d* = 1.4	*d* = 3.0	*d* = 0.9
		*p* < 0.001	*p* < 0.001	*p* < 0.05
	Right ear (IC)	*d* = 1.6	*d* = 2.4	*d* = 0.2
		*p* < 0.001	*p* < 0.001	TD

*Cohen's d and p-value were reported for different pairs of age cohorts. For 80 Hz, the results were also reported for laterality index of the medial geniculate body (MGB) and inferior colliculus (IC). The trivial difference is indicated by TD and refers to when Cohen's d suggested a small (d ≤0.2) effect size of mean differences (Sullivan and Feinn, 2012), and NS goes for not significant*.

The hemispheric asymmetry was also investigated for the subcortical sources of 80 Hz due to the importance of these activations in 80 Hz (Figure 5B). In these neural sources, a reduction in asymmetry in older participants was observed for stimuli presented to the right side.

Briefly, altered hemispheric asymmetry in older and middle-aged participants was observed in all stimulation conditions. However, the patterns of age-related changes were variable across different stimulation conditions.

## Discussion

### Effect of Age on the Activation of Neural Generators

The current study shows that age affects neural generators, albeit to a different extent for those responding to slow or fast modulations. The effect of age on the ASSRs was investigated for different modulation frequencies and categories of neural generators in persons with normal audiometric thresholds to avoid HL as a confounder as much as possible.

Considering all ROIs, older participants exhibited enhanced ASSRs compared to young and middle-aged participants for slow AM modulations (<50 Hz). These modulation frequencies are similar to the repetition rate of phonemes and syllables in speech. This age effect was also observed in different categories of sources, except for non-primary sources at 20 Hz modulation frequency. Our results on low-frequency ASSRs, particularly 4 Hz ASSRs, are in line with those of the study by Tlumak et al. (2015) who showed that 5 Hz ASSRs were larger in older listeners compared to young participants. However, no age-related changes in 20 Hz ASSRs have been reported in sensor-level studies (Leigh-Paffenroth and Fowler, 2006; Tlumak et al., 2015, Goossens et al., 2016).

The effect of age on 40 Hz ASSRs has been investigated before. While sensor-level analysis did not report significant changes across age (Boettcher et al., 2001; Leigh-Paffenroth and Fowler, 2006; Goossens et al., 2016), a source-level analysis reported a tendency for increased ASSR amplitudes in response to 40 Hz AM stimuli for adults aged between 19 and 45 years (Poulsen et al., 2007). The age effect was only observed for the dipole in the left hemisphere, while they used three dipoles (left/right hemisphere and the brain stem) for source modeling. The inconsistency between Poulsen's study and ours can be due to the different age ranges of the participants and/or the prior assumption about the number of sources. Modeling brain responses with a limited number of dipoles less than the actual number of generators may reduce sensitivity to changes of neural activities. This reduced sensitivity can also be seen in sensor-level data, which show a linear combination of neural activities.

For fast AM modulations (80 Hz), the ASSR activities of neural generators in the middle-aged and older adults were less than those of the younger persons. A similar age effect was also observed for other categories of sources except when comparing the cortical generators of young and older groups. These results are in line with previous EEG studies (Dimitrijevic et al., 2004; Purcell et al., 2004; Leigh-Paffenroth and Fowler, 2006) and also with animal studies (Parthasarathy et al., 2010; Herrmann et al., 2017). Potential mechanisms underlying these age-related changes will be explained further in *Potential Mechanisms Underlying Age-Related Changes in Temporal Processing*.

Interestingly, speech intelligibility data of the same young, middle-aged, and older participants show that speech perception declines by middle age and decreases further onto older age even when hearing sensitivity is clinically normal and there is no indication of mild cognitive impairment (Goossens et al., 2017). These differences in speech understanding could well be the functional consequences of the observed changes in neural synchronization.

### Effect of Age on Phase-Locking of Neural Generators

Comparison of phase coherence across age suggests a decline of phase-locking in older and middle-aged adults compared to young participants in response to 80 Hz AM stimuli. This age effect was also observed in different categories of sources, with the exception for young and middle-aged persons in a cortical category of sources. For slow modulation frequencies (<50 Hz), no significant effect of age was observed when considering all sources. However, a small age effect was observed in a few categories of sources (for example, cortical sources at 4 Hz).

Very few studies investigated phase-locking independently of ASSR amplitude. Leigh-Paffenroth and Fowler (2006) reported fewer phase-locked responses at 90 Hz modulation rate for older adults compared to young, while the differences in the number of responses at 20 and 40 Hz were not significant. This age-related change is also observed in the present study. In another study, Edgar et al. (2017) suggested an association between the phase coherence of 40 Hz ASSR and age (20–60 years) in the left hemisphere, not in the right one.

Our results concerning the age-related changes in phase-locking are also consistent with event-related potential (ERP) studies, which observed a reduction in phase coherence of auditory brain stem responses to tonal and speech stimuli (Clinard et al., 2010; Anderson et al., 2012; Clinard and Tremblay, 2013; Presacco et al., 2015; Roque et al., 2019). In addition, our results are also consistent with animal studies, where less phase-locking to rapid amplitude modulations has been reported in near- and far-field recordings (Parthasarathy and Bartlett, 2012; Herrmann et al., 2017; Parthasarathy et al., 2019).

### Potential Mechanisms Underlying Age-Related Changes in Temporal Processing

Decrease of the GABAergic and glycinergic inhibitory neurotransmission is a consistent finding across central auditory regions of aging animals (Ling et al., 2005; Frisina and Walton, 2006; Caspary et al., 2008, 2013; Parthasarathy et al., 2010; Herrmann et al., 2017), leading to a downregulation in functional inhibition. Loss of normal functional inhibition has also been reported in humans (Chen et al., 2013) and may lead to increased spontaneous and sound-evoked discharged rates (Hughes et al., 2010; Parthasarathy et al., 2014, 2019; Herrmann et al., 2017).

Inhibitory neurotransmission also plays a key role in shaping the response to complex and/or rapid temporally modulated stimuli (Walton et al., 1998; Caspary et al., 2008; Parthasarathy et al., 2010). Reduction in inhibitory neurotransmission in older adults may result in a loss of temporal precision in encoding rapidly changing sounds (Anderson et al., 2012; Roque et al., 2019).

The non-significant change of phase coherence across age for neural generators in response to slow acoustic modulations (<40 Hz) suggests that age does not affect the ability to follow slow envelopes. In addition, the increased ASSR amplitude for older participants in this frequency range is in line with the increased neural excitability (central gain) as a consequence of the loss of inhibition (Chambers et al., 2016; Herrmann et al., 2017). However, we observed a decline in response strength and phase-locking to fast acoustic modulations with advancing age. This finding is in line with the decreased temporal precision in following the rapid modulated stimuli due to the loss of functional inhibition (Anderson et al., 2012).

In sum, our results demonstrated age-related changes in the amplitude and the phase coherence of ASSRs, which can be interpreted as changes in the neural excitability and the phase-locking ability of the central auditory system, respectively. These age-related changes in temporal processing of slow and fast acoustic modulations are in line with the expected consequences of the loss of functional inhibition across the central auditory system with increasing age.

### Age-Related Changes in Neural Dynamics at Middle Age

Our results indicated meaningful age-related changes in the phase locking and response strength in the middle-aged cohort compared to the young one, specifically in response to 80 Hz acoustic modulations. In line with previous studies, these findings suggest that the age-related changes in temporal processing are already apparent in the middle agers (Ross et al., 2007; Ross, 2008, Leigh-Paffenroth and Elangovan, 2011). In a behavioral study on the same data as here, a meaningful decline in speech perception performance was also observed in the middle-aged cohort (Goossens et al., 2017).

Our data highlight the importance of auditory screening (e.g., speech-in-noise test) at midlife, where changes already appear to occur. A recent model presented by the Lancet Commission on Dementia Prevention, Intervention and Care shows that hearing impairment is the largest potentially modifiable risk factor for dementia among nine health and lifestyle factors (Livingston et al., 2017). Strikingly, the model shows that midlife hearing impairment, if eliminated, might reduce the risk of dementia by 9%. Furthermore, our findings suggest that training/rehabilitating fast acoustic modulations where results showed a reduced temporal precision via decreased phase-locking and smaller response magnitudes might facilitate perception. However, more research is needed to clarify different age-related changes that may cause poorer speech perception in the elderly.

### Hemispheric Asymmetry

#### Hemispheric Asymmetry in a Young Cohort

Overall, our results revealed that by varying the modulation frequency and side of stimulation, hemispheric asymmetry changes significantly within the young cohort. This finding is generally consistent with previous studies and emphasizes the importance of modulation frequency and side of stimulation when studying temporal envelope processing (Ross et al., 2005).

In the present study, for 4 and 20 Hz AM stimuli, contralateral and ipsilateral activations in the AC were observed, respectively, which are in line with functional MRI (fMRI) observations (Langers et al., 2005) and with previous source-level EEG studies (Luke et al., 2017). An asymmetry to the left AC was observed for 40 Hz modulation frequency, left and right stimulation. These results are not consistent with the previous source-level EEG studies (Edgar et al., 2017; Luke et al., 2017). For 80 Hz AM stimuli presented to the left or right ear, right hemispheric asymmetry was observed for subcortical activations except that for CN at left side stimulation. To the best of our knowledge, no other study has investigated lateralization of subcortical sources in response to fast AM stimuli. While the contralateral responses are well-known for different sensory modalities (Del Gratta et al., 2002; Hemond et al., 2007) and also for transient auditory responses (Ross et al., 2005), the abovementioned results suggest that hemispheric asymmetry of ASSRs is not contralateral for all different modulation rates. This finding is in line with that of previous studies showing that the laterality of sustained responses like ASSRs can be distinct from that of transient responses (Ross et al., 2005; Lehmann et al., 2007). Moreover, these results show that laterality for ASSRs is sensitive to stimulus periodicity. We found contralateral asymmetry for 4Hz ASSR and ipsilateral asymmetry for 20 Hz ASSR for the young cohort.

We did not compare our results with those of sensor-level studies because sensor-level asymmetry depends highly on the configuration of the neural sources relative to the EEG electrodes and can be completely opposite to the source-level asymmetry. For example, source-level EEG studies and the fMRI observations suggest left hemisphere asymmetry for 4 Hz AM stimuli presented to the right ear (Langers et al., 2005; Luke et al., 2017), while sensor-level studies suggest right hemisphere lateralization (Poelmans et al., 2012; Vanvooren et al., 2014). To avoid this kind of bias, which is mainly due to the volume conduction problem in the sensor-level approach, it is important to perform source modeling before investigating laterality. Moreover, among the source-level studies, the prior assumptions of the source modeling about the number of sources can exert an influence on the LI. For example, in a separate analysis, we fitted two dipoles to the 40 Hz ASSRs and obtained a right hemispheric asymmetry, which is in line with previous source-level studies with two dipoles in the left and right AC (Ross et al., 2005; Edgar et al., 2017). Nevertheless, in the current study using the MNI approach, with minimal restrictions regarding the number and location of the sources, we found a left hemisphere lateralization on the same data. Thus, the prior assumptions of the source analysis approach should be taken into account before comparing the results of different studies.

#### Effect of Age on Hemispheric Asymmetry

In general, our results suggest that aging occurs with the altered hemispheric asymmetry in auditory temporal processing. This finding is in line with the previous studies suggesting that the altered hemispheric processing patterns might be a reason for the impaired speech processing in older adults or persons with dyslexia (Goswami, 2011; Vanvooren et al., 2014; Goossens et al., 2016).

A wide range of neurophysiological (inhibition reduction) and anatomical changes (cerebral atrophy, demyelination) associated with aging has been reported in several studies (Giroud et al., 2018, 2019). As we discussed earlier, these changes possibly underlie the altered neural responses in the older population and can differ for different modulation rates. Importantly, these changes (neurophysiological and anatomical) may vary across brain regions or across hemispheres (Chen et al., 2013) and may lead to an altered hemispheric asymmetry in older adults. In a theoretical form, the hemispheric asymmetry could be estimated by knowing the effect of neurophysiological and anatomical changes due to aging for each hemisphere separately.

Up to now, only a few studies have investigated the effect of age on the hemispheric asymmetry of low-level auditory processing such as temporal envelope processing. For ***4 Hz*** ASSRs with left side stimulation, the LI of AC is positive in the young group and decreased in older adults. For the right side stimulation, the negative LI of the young group gradually increased (became more negative) across age. These two observations suggest that the hemispheric asymmetry of 4 Hz ASSR is moved toward the left hemisphere for both the left and right side of stimulation. However, within the framework of “asymmetric sampling in time” (AST) hypothesis, it has been shown that in the normal listeners, the slowly changing speech features (unfolded in a longer timescale of about ~250 ms) related to syllables are preferentially processed by the right auditory-related areas (Poeppel, 2003; Shalom and Poeppel, 2008; etc.). Taking the AST hypothesis into account, this age-related change of lateralization (moving toward the left hemisphere) can be a possible reason for the impaired speech perception in older people.

In response to ***20 Hz***AM stimuli presented to the left ear, the hemispheric asymmetry gradually decreased across age. For ***80 Hz***ASSRs at both sides of stimulation, a right hemispheric asymmetry was observed for subcortical sources (except for CN at left side stimulation) of young adults, while symmetrical responses were found for subcortical sources of older adults. The pattern of age-related alternations in 20 and 80 Hz is similar to that already reported for pre-frontal activity during cognitive control of semantic and working memory and perception (Cabeza et al., 2002). Our finding regarding a reduction of hemispheric asymmetry for low-level auditory processing (i.e., in response to 20 and 80 Hz AM stimuli) may suggest a more global regime of reduced asymmetry in the aging population.

In the current study, we corrected for multiple comparisons and provided effect sizes; however, the large number of comparisons in relatively small groups of participants may lead to a Type I error from random statistical variation. To control for this potential error, it would be appropriate to replicate this study for a different data set.

### The Effect of Source-Level Analysis

Due to the volume conduction of brain tissue, a linear mixture of neural activity of different sources is recorded in scalp-level measurements. Via source modeling, we resolve the mixture and investigate the activity of the neural sources, separately, in different age cohorts. The source-level analysis is also beneficial to detect small age effects on a neural source, which may be hidden by other generator's activity in the scalp-level analysis. For instance, we found increased response strengths to 20 and 40 Hz AM stimuli, while no changes were observed using sensor-level analyses on the same data (Goossens et al., 2016). Note that the age-related changes in 4 and 80 Hz ASSRs were similar for source-level and sensor-level analyses.

Volume conduction also has a remarkable influence on hemispheric asymmetry. The sensor-level asymmetry depends on the activity and location of several neural generators. Indeed, this asymmetry can be different from the asymmetry of each individual neural generator. For example, scalp-level studies suggest right hemisphere lateralization for 4 Hz AM stimuli presented to the right ear (Poelmans et al., 2012; Vanvooren et al., 2014), while source-level EEG studies and the fMRI observations suggest left hemisphere asymmetry (Langers et al., 2005; Luke et al., 2017). As a result, it seems that a source-level analysis provides a more sensitive framework than a sensor-level one. It should be noted that certain choices regarding parameters, such as the head model, the electrical conductance of brain tissues, and type of optimization for the inverse problem, may influence the results of the source reconstruction. However, since the same methodology was used for different age cohorts, the comparisons and the conclusions drawn from them appear well-grounded.

## Conclusion

The present study investigated the effect of age on the neural generators involved in the temporal envelope processing in adults with normal audiometric thresholds. A wide range of neural generators of ASSRs in response to 4, 20, 40, and 80 Hz acoustic modulations was reconstructed in young, middle-aged, and older participants. Age-related changes were observed for response strength, phase coherence, and hemispheric asymmetry. For slow acoustic modulations (below 50 Hz), the response amplitudes were higher in older participants than young ones, while the phase coherences were almost similar for the three age cohorts. For fast acoustic modulations, both the response amplitudes and phase coherences were reduced in older participants compared to younger and middle-aged persons. The observed age-related changes of neural activations in response to both slow and fast acoustic modulations can be explained by the loss of functional inhibition in older adults (Caspary et al., 2008). Older persons also demonstrate altered hemispheric processing, which, in turn, may impact their speech processing. An important finding was that the abovementioned age-related changes are already apparent in the middle agers well before observable HL is noted.

## Data Availability Statement

The data that support the findings of this study are available on request from the corresponding author. The data are not publicly available due to privacy/ethical restrictions (containing information that could compromise the privacy of research participants).

## Ethics Statement

The studies involving human participants were reviewed and approved by Medical Ethical Committee of the University Hospitals and University of Leuven (approval number B322201214866. The patients/participants provided their written informed consent to participate in this study.

## Author Contributions

EF, JW, and AW designed the study. EF analyzed data and performed statistical analyses. JW, and AW verified the analytical methods and supported data analysis. EF, JW, and AW contributed to the interpretation of the results. EF wrote the manuscript draft. EF, JW, and AW critically revised the manuscript. All authors contributed to the article and approved the submitted version.

## Conflict of Interest

The authors declare that the research was conducted in the absence of any commercial or financial relationships that could be construed as a potential conflict of interest.

## References

[B1] Anderson S. Parbery-Clark A. White-Schwoch T. Kraus N. (2012). Aging affects neural precision of speech encoding. J. Neurosci. 32, 14156–14164. 10.1523/JNEUROSCI.2176-12.2012 23055485 PMC3488287

[B2] Benjamini Y. Hochberg Y. (1995). Controlling the false discovery rate: a practical and powerful approach to multiple testing. J. R. Stat. Soc. Ser. B 57, 289–300. 10.1111/j.2517-6161.1995.tb02031.x

[B3] Berlingeri M. Danelli L. Bottini G. Sberna M. Paulesu E. (2013). Reassessing the HAROLD model: is the hemispheric asymmetry reduction in older adults a special case of compensatory-related utilisation of neural circuits? Exp. Brain Res. 224, 393–410. 10.1007/s00221-012-3319-x 23178904

[B4] Boettcher F. A. Poth E. A. Mills J. H. Dubno J. R. (2001). The amplitude-modulation following response in young and aged human subjects. Hear. Res. 153, 32–42. 10.1016/S0378-5955(00)00255-0 11223295

[B5] Bradley A. Yao J. Dewald J. Richter C. P. (2016). Evaluation of electroencephalography source localization algorithms with multiple cortical sources. PLoS ONE 11:e0147266. 10.1371/journal.pone.0147266 26809000 PMC4725774

[B6] Cabeza R. (2002). Hemispheric asymmetry reduction in older adults: the HAROLD model. Psychol. Aging 17, 85–100. 10.1037/0882-7974.17.1.85 11931290

[B7] Cabeza R. Anderson N. D. Locantore J. K. McIntosh A. R. (2002). Aging gracefully: compensatory brain activity in high-performing older adults. Neuroimage 17, 1394–1402. 10.1006/nimg.2002.1280 12414279

[B8] Caspary D. M. Hughes L. F. Ling L. L. (2013). Age-related GABAA receptor changes in rat auditory cortex. Neurobiol. Aging 34, 1486–1496. 10.1016/j.neurobiolaging.2012.11.009 23257264 PMC3570724

[B9] Caspary D. M. Ling L. Turner J. G. Hughes L. F. (2008). Inhibitory neurotransmission, plasticity and aging in the mammalian central auditory system. J. Exp. Biol. 211, 1781–1791. 10.1242/jeb.013581 18490394 PMC2409121

[B10] Chambers A. R. Resnik J. Yuan Y. Whitton J. P. Edge A. S. Liberman M. C.. (2016). Central gain restores auditory processing following near-complete cochlear denervation. Neuron 89, 867–879. 10.1016/j.neuron.2015.12.041 26833137 PMC4760846

[B11] Chen J. L. Ros T. Gruzelier J. H. (2013). Dynamic changes of ICA-derived EEG functional connectivity in the resting state. Hum. Brain Mapp. 34, 852–868. 10.1002/hbm.21475 22344782 PMC6870341

[B12] Clinard C. G. Tremblay K. L. (2013). Aging degrades the neural encoding of simple and complex sounds in the human brainstem. J. Am. Acad. Audiol. 24, 590–599. 10.3766/jaaa.24.7.7 24047946

[B13] Clinard C. G. Tremblay K. L. Krishnan A. R. (2010). Aging alters the perception and physiological representation of frequency: evidence from human frequency-following response recordings. Hear. Res. 264, 48–55. 10.1016/j.heares.2009.11.010 19944140 PMC2868068

[B14] Coffey E. B. J. Herholz S. C. Chepesiuk A. M. P. Baillet S. Zatorre R. J. (2016). Cortical contributions to the auditory frequency-following response revealed by MEG. Nat. Commun. 7:11070. 10.1038/ncomms11070 27009409 PMC4820836

[B15] Cogan G. B. Poeppel D. (2011). A mutual information analysis of neural coding of speech by low-frequency MEG phase information. J. Neurophysiol. 106, 554–563. 10.1152/jn.00075.2011 21562190 PMC3154802

[B16] Cohen B. H. (2002). Calculating a factorial ANOVA from means and standard deviations. Underst. Stat. 1, 191–203. 10.1207/S15328031US0103_04

[B17] Cohen J. (1988). Statistical Power Analysis for the Behavioral Sciences, 2nd Edn. New York, NY: Routledge.

[B18] Dale A. M. Liu A. K. Fischl B. R. Buckner R. L. Belliveau J. W. Lewine J. D.. (2000). Neurotechnique mapping : combining fMRI and MEG for high-resolution imaging of cortical activity. Neuron 26, 55–67. 10.1016/S0896-6273(00)81138-1 10798392

[B19] Del Gratta C. Della Penna S. Ferretti A. Franciotti R. Pizzella V. Tartaro A.. (2002). Topographic organization of the human primary and secondary somatosensory cortices: comparison of fMRI and MEG findings. Neuroimage 17, 1373–1383. 10.1006/nimg.2002.1253 12414277

[B20] Desikan R. S. Ségonne F. Fischl B. Quinn B. T. Dickerson B. C. Blacker D.. (2006). An automated labeling system for subdividing the human cerebral cortex on MRI scans into gyral based regions of interest. Neuroimage 31, 968–980. 10.1016/j.neuroimage.2006.01.021 16530430

[B21] Dimitrijevic A. John M. S. Picton T. W. (2004). Auditory steady-state responses and word recognition scores in normal-hearing and hearing-impaired adults. Ear Hear. 25, 68–84. 10.1097/01.AUD.0000111545.71693.48 14770019

[B22] Dobie R. A. Wilson M. J. (1996). A comparison of ttest, Ftest, and coherence methods of detecting steady-state auditory-evoked potentials, distortion-product otoacoustic emissions, or other sinusoids. J. Acoust. Soc. Am. 100, 2236–2246. 10.1121/1.417933 8865632

[B23] Drullman R. Festen J. M. Plomp R. (1994). Effect of reducing slow temporal modulations on speech reception. J. Acoust. Soc. Am. 95, 2670–2680. 10.1121/1.409836 8207140

[B24] Du Y. Buchsbaum B. R. Grady C. L. Alain C. (2016). Increased activity in frontal motor cortex compensates impaired speech perception in older adults. Nat. Commun. 712241. 10.1038/ncomms12241 27483187 PMC4974649

[B25] Edgar J. C. Fisk C. L IV. Chen Y. H. Stone-Howell B. Hunter M. A. Huang M.. (2017). By our bootstraps: comparing methods for measuring auditory 40 Hz steady-state neural activity. Psychophysiology 54, 1110–1127. 10.1111/psyp.12876 28421620 PMC5507710

[B26] Efron B. Stein C. (1981). The Jackknife estimate of variance. Ann. Stat. 9, 586–596. 10.1214/aos/1176345462

[B27] Farahani E. D. Goossens T. Wouters J. van Wieringen A. (2017). Spatiotemporal reconstruction of auditory steady-state responses to acoustic amplitude modulations: potential sources beyond the auditory pathway. Neuroimage 148, 240–253. 10.1016/j.neuroimage.2017.01.032 28110090

[B28] Farahani E. D. Wouters J. van Wieringen A. (2019). Contributions of non-primary cortical sources to auditory temporal processing. Neuroimage 191, 303–314. 10.1016/j.neuroimage.2019.02.037 30794868

[B29] Farahani E. D. Wouters J. van Wieringen A. (2020). Brain mapping of auditory steady-state responses: a broad view of cortical and subcortical sources. Hum. Brain Mapp. 1–17. 10.1002/hbm.25262 33166050 PMC7814770

[B30] Fonov V. Evans A. C. Botteron K. Almli C. R. McKinstry R. C. Collins D. L. (2011). Unbiased average age-appropriate atlases for pediatric studies. Neuroimage 54, 313–327. 10.1016/j.neuroimage.2010.07.033 20656036 PMC2962759

[B31] Frisina R. D. Walton J. P. (2006). Age-related structural and functional changes in the cochlear nucleus. Hear. Res. 216–217, 216–223. 10.1016/j.heares.2006.02.003 16597491

[B32] Ghumare E. G. Schrooten M. vandenberghe R. Dupont P. (2018). A time-varying connectivity analysis from distributed EEG sources: a simulation study. Brain Topogr. 31, 721–737. 10.1007/s10548-018-0621-3 29374816 PMC6097773

[B33] Giraud A. Lorenzi C. Ashburner J. Wable J. Johnsrude I. Frackowiak R.. (2000). Representation of the temporal envelope of sounds in the human brain. J. Neurophysiol. 84, 1588–1598. 10.1152/jn.2000.84.3.1588 10980029

[B34] Giroud N. Keller M. Hirsiger S. Dellwo V. Meyer M. (2019). Bridging the brain structure—brain function gap in prosodic speech processing in older adults. Neurobiol. Aging 80, 116–126. 10.1016/j.neurobiolaging.2019.04.017 31170532

[B35] Giroud N. Lemke U. Reich P. Bauer J. Widmer S. Meyer M. (2018). Are you surprised to hear this? Longitudinal spectral speech exposure in older compared to middle-aged normal hearing adults. Eur. J. Neurosci. 47, 58–68. 10.1111/ejn.13772 29119612

[B36] Goossens T. Vercammen C. Wouters J. van Wieringen A. (2016). Aging affects neural synchronization to speech-related acoustic modulations. Front. Aging Neurosci. 8:133. 10.3389/fnagi.2016.00133 27378906 PMC4908923

[B37] Goossens T. Vercammen C. Wouters J. van Wieringen A. (2017). Masked speech perception across the adult lifespan: impact of age and hearing impairment. Hear. Res. 344, 109–124. 10.1016/j.heares.2016.11.004 27845259

[B38] Goossens T. Vercammen C. Wouters J. van Wieringen A. (2019). The association between hearing impairment and neural envelope encoding at different ages. Neurobiol. Aging 74, 202–212. 10.1016/j.neurobiolaging.2018.10.008 30472387

[B39] Goswami U. (2011). A temporal sampling framework for developmental dyslexia. Trends Cogn. Sci. 15, 3–10. 10.1016/j.tics.2010.10.001 21093350

[B40] Gramfort A. Papadopoulo T. Olivi E. Clerc M. (2010). OpenMEEG: opensource software for quasistatic bioelectromagnetics. Biomed. Eng. Online 9:45. 10.1186/1475-925X-9-45 20819204 PMC2949879

[B41] Greenwald R. R. Jerger J. (2001). Aging affects hemispheric asymmetry on a competing speech task. J. Am. Acad. Audiol. 12, 167–173. 11332516

[B42] Hämäläinen J. A. Rupp A. Soltész F. Szücs D. Goswami U. (2012). Reduced phase locking to slow amplitude modulation in adults with dyslexia: an MEG study. Neuroimage 59, 2952–2961. 10.1016/j.neuroimage.2011.09.075 22001790

[B43] Hauk O. Wakeman D. G. Henson R. (2011). Comparison of noise-normalized minimum norm estimates for MEG analysis using multiple resolution metrics. Neuroimage 54, 1966–1974. 10.1016/j.neuroimage.2010.09.053 20884360 PMC3018574

[B44] He B. Sohrabpour A. Brown E. Liu Z. (2018). Electrophysiological source imaging: a noninvasive window to brain dynamics. Annu. Rev. Biomed. Eng. 20, 171–196. 10.1146/annurev-bioeng-062117-120853 29494213 PMC7941524

[B45] Hemond C. C. Kanwisher N. G. Op de Beeck H. P. (2007). A preference for contralateral stimuli in human object- and face-selective cortex. PLoS ONE 2:e574. 10.1371/journal.pone.0000574 17593973 PMC1894654

[B46] Herdman A. T. Lins O. Van Roon P. Stapells D. R. Scherg M. Picton T. W. (2002). Intracerebral sources of human auditory steady-state responses. Brain Topogr. 15, 69–86. 10.1023/A:1021470822922 12537303

[B47] Herrmann B. Parthasarathy A. Bartlett E. L. (2017). Ageing affects dual encoding of periodicity and envelope shape in rat inferior colliculus neurons. Eur. J. Neurosci. 45, 299–311. 10.1111/ejn.13463 27813207 PMC5247336

[B48] Hincapié A. S. Kujala J. Mattout J. Daligault S. Delpuech C. Mery D.. (2016). MEG connectivity and power detections with minimum norm estimates require different regularization parameters. Comput. Intell. Neurosci. 2016:3979547. 10.1155/2016/3979547 27092179 PMC4820599

[B49] Hughes L. F. Turner J. G. Parrish J. L. Caspary D. M. (2010). Processing of broadband stimuli across A1 layers in young and aged rats. Hear. Res. 264, 79–85. 10.1016/j.heares.2009.09.005 19772906 PMC2868092

[B50] International Organization for Standardization (2000). ISO-7029: Acoustics Statistical Distribution of Hearing Thresholds as a Function of Age. Geneva: International Organization for Standardization.

[B51] John M. S. Picton T. W. (2000). Human auditory steady-state responses to amplitude-modulated tones phase and latency measurements. Hear. Res. 141, 57–79. 10.1016/S0378-5955(99)00209-9 10713496

[B52] Kang S. S. Lano T. J. Sponheim S. R. (2015). Distortions in EEG interregional phase synchrony by spherical spline interpolation: causes and remedies. Neuropsychiatr. Electrophysiol. 1:9 10.1186/s40810-015-0009-5

[B53] Koerner T. K. Zhang Y. (2015). Effects of background noise on inter-trial phase coherence and auditory N1-P2 responses to speech stimuli. Hear. Res. 328, 113–119. 10.1016/j.heares.2015.08.002 26276419

[B54] Langers D. R. M. Van Dijk P. Backes W. H. (2005). Lateralization, connectivity and plasticity in the human central auditory system. Neuroimage 28, 490–499. 10.1016/j.neuroimage.2005.06.024 16051500

[B55] Lehmann C. Herdener M. Schneider P. Federspiel A. Bach D. R. Esposito F.. (2007). Dissociated lateralization of transient and sustained blood oxygen level-dependent signal components in human primary auditory cortex. Neuroimage 34, 1637–1642. 10.1016/j.neuroimage.2006.11.011 17175176

[B56] Leigh-Paffenroth E. D. Elangovan S. (2011). Temporal processing in low-frequency channels: effects of age and hearing loss in middle-aged listeners. J. Am. Acad. Audiol. 22, 393–404. 10.3766/jaaa.22.7.2 21993047

[B57] Leigh-Paffenroth E. D. Fowler C. G. (2006). Amplitude-modulated auditory steady-state responses in younger and older listeners. J. Am. Acad. Audiol. 17, 582–597. 10.3766/jaaa.17.8.5 16999253

[B58] Liégeois-Chauvel C. Lorenzi C. Trébuchon A. Régis J. Chauvel P. (2004). Temporal envelope processing in the human left and right auditory cortices. Cereb. Cortex 14, 731–740. 10.1093/cercor/bhh033 15054052

[B59] Lin F. H. Witzel T. Ahlfors S. P. Stufflebeam S. M. Belliveau J. W. Hämäläinen M. S. (2006). Assessing and improving the spatial accuracy in MEG source localization by depth-weighted minimum-norm estimates. Neuroimage 31, 160–171. 10.1016/j.neuroimage.2005.11.054 16520063

[B60] Ling L. L. Hughes L. F. Caspary D. M. (2005). Age-related loss of the GABA synthetic enzyme glutamic acid decarboxylase in rat primary auditory cortex. Neuroscience 132, 1103–1113. 10.1016/j.neuroscience.2004.12.043 15857714

[B61] Livingston G. Sommerlad A. Orgeta V. Costafreda S. G. Huntley J. Ames D.. (2017). Dementia prevention, intervention, and care. Lancet 390, 2673–2734. 10.1016/S0140-6736(17)31363-6 28735855

[B62] Luke R. De Vos A. Wouters J. (2017). Source analysis of auditory steady-state responses in acoustic and electric hearing. Neuroimage 147, 568–576. 10.1016/j.neuroimage.2016.11.023 27894891

[B63] Luo H. Poeppel D. (2007). Phase patterns of neuronal responses reliably discriminate speech in human auditory cortex. Neuron 54, 1001–1010. 10.1016/j.neuron.2007.06.004 17582338 PMC2703451

[B64] Millman R. E. Mattys S. L. Gouws A. D. Prendergast G. (2017). Magnified neural envelope coding predicts deficits in speech perception in noise. J. Neurosci. 37, 7727–7736. 10.1523/JNEUROSCI.2722-16.2017 28694336 PMC5551064

[B65] Nagy P. (2013). n-way ANOVA from summary statistics. MATLAB Cent. File Exch. Available online at: https://www.mathworks.com/matlabcentral/fileexchange/41036-n-way-anova-from-summary-statistics (Retrieved September 11, 2018).

[B66] Nasreddine Z. Phillips N. Bedirian V. Charbonneau S. Whitehead V. Collin I. (2005). The montreal cognitive assessment, MoCA : a brief screening. J. Am. Geriatr. Soc. 53, 695–699. 10.1111/j.1532-5415.2005.53221.x 15817019

[B67] Oldfield R. (1971). The assessment and analysis of handedness. Neuropsychologia 9, 97–113. 10.1016/0028-3932(71)90067-4 5146491

[B68] Oostenveld R. Fries P. Maris E. Schoffelen J. M. (2011). FieldTrip: open source software for advanced analysis of MEG, EEG, and invasive electrophysiological data. Comput. Intell. Neurosci. 2011:156869. 10.1155/2011/156869 21253357 PMC3021840

[B69] Ostroff J. M. McDonald K. L. Schneider B. A. Alain C. (2003). Aging and the processing of sound duration in human auditory cortex. Hear. Res. 181, 1–7. 10.1016/S0378-5955(03)00113-8 12855356

[B70] Overath T. Zhang Y. Sanes D. H. Poeppel D. (2012). Sensitivity to temporal modulation rate and spectral bandwidth in the human auditory system : fMRI evidence sensitivity to temporal modulation rate and spectral bandwidth in the human auditory system : fMRI evidence. J. Neurophysiol. 107, 2042–2056. 10.1152/jn.00308.2011 22298830 PMC3331610

[B71] Parthasarathy A. Bartlett E. (2012). Two-channel recording of auditory-evoked potentials to detect age-related deficits in temporal processing. Hear. Res. 289, 52–62. 10.1016/j.heares.2012.04.014 22560961 PMC3371184

[B72] Parthasarathy A. Cunningham P. A. Bartlett E. L. (2010). Age-related differences in auditory processing as assessed by amplitude-modulation following responses in quiet and in noise. Front. Aging Neurosci. 2:152. 10.3389/fnagi.2010.00152 21188162 PMC3006655

[B73] Parthasarathy A. Datta J. Torres J. A. L. Hopkins C. Bartlett E. L. (2014). Age-related changes in the relationship between auditory brainstem responses and envelope-following responses. J. Assoc. Res. Otolaryngol. 15, 649–661. 10.1007/s10162-014-0460-1 24845405 PMC4141432

[B74] Parthasarathy A. Herrmann B. Bartlett E. L. (2019). Aging alters envelope representations of speech-like sounds in the inferior colliculus. Neurobiol. Aging 73, 30–40. 10.1016/j.neurobiolaging.2018.08.023 30316050 PMC6251750

[B75] Peelle J. E. Davis M. H. (2012). Neural oscillations carry speech rhythm through to comprehension. Front. Psychol. 3:320. 10.3389/fpsyg.2012.00320 22973251 PMC3434440

[B76] Perrin F. Pernier J. Bertrand O. (1989). Spherical splines for scalp potential and current density mapping. Clin. Neurophysiol. 72, 184–187. 10.1016/0013-4694(89)90180-6 2464490

[B77] Picton T. (2013). Hearing in time: evoked potential studies of temporal processing. Ear Hear. 34, 385–401. 10.1097/AUD.0b013e31827ada02 24005840

[B78] Picton T. W. Dimitrijevic A. Perez-Abalo M.-C. Van Roon P. (2005). Estimating audiometric thresholds using auditory steady-state responses. J. Am. Acad. Audiol. 16, 140–156. 10.3766/jaaa.16.3.3 15844740

[B79] Picton T. W. Dimitrijevic A. Sasha John M. Van Roon P. (2001). The use of phase in the detection of auditory steady-state responses. Clin. Neurophysiol. 112, 1698–1711. 10.1016/S1388-2457(01)00608-3 11514253

[B80] Poelmans H. Luts H. Vandermosten M. Ghesquière P. Wouters J. (2012). Hemispheric asymmetry of auditory steady-state responses to monaural and diotic stimulation. J. Assoc. Res. Otolaryngol. 13, 867–876. 10.1007/s10162-012-0348-x 22926721 PMC3505592

[B81] Poeppel D. (2003). The analysis of speech in different temporal integration windows: cerebral lateralization as “asymmetric sampling in time.” Speech Commun. 41, 245–255. 10.1016/S0167-6393(02)00107-3

[B82] Poulsen C. Picton T. W. Paus T. (2007). Age-related changes in transient and oscillatory brain responses to auditory stimulation in healthy adults 19-45 years old. Cereb. Cortex 17, 1454–1467. 10.1093/cercor/bhl056 16916887

[B83] Presacco A. Jenkins K. Lieberman R. Anderson S. (2015). Effects of aging on the encoding of dynamic and static components of speech. Ear Hear. 36, e352–e363. 10.1097/AUD.0000000000000193 26177213 PMC4839261

[B84] Presacco A. Simon J. Z. Anderson S. (2019). Speech-in-noise representation in the aging midbrain and cortex: effects of hearing loss. PLoS ONE 14:e01213899. 10.1371/journal.pone.0213899 30865718 PMC6415857

[B85] Purcell D. W. John S. M. Schneider B. A. Picton T. W. (2004). Human temporal auditory acuity as assessed by envelope following responses. J. Acoust. Soc. Am. 116, 3581–3593. 10.1121/1.1798354 15658709

[B86] Roque L. Karawani H. Gordon-Salant S. Anderson S. (2019). Effects of age, cognition, and neural encoding on the perception of temporal speech cues. Front. Neurosci. 13:749. 10.3389/2fnins.2019.00749 31379494 PMC6659127

[B87] Rosen S. (1992). Temporal information in speech: acoustic, auditory and linguistic aspects. Philos. Trans. Biol. Sci. 336, 367–373. 10.1098/rstb.1992.0070 1354376

[B88] Ross B. (2008). A novel type of auditory responses: temporal dynamics of 40-hz steady-state responses induced by changes in sound localization. J. Neurophysiol. 100, 1265–1277. 10.1152/jn.00048.2008 18632891

[B89] Ross B. Fujioka T. Tremblay K. L. Picton T. W. (2007). Aging in binaural hearing begins in mid-life: evidence from cortical auditory-evoked responses to changes in interaural phase. J. Neurosci. 27, 11172–11178. 10.1523/JNEUROSCI.1813-07.2007 17942712 PMC6673023

[B90] Ross B. Herdman A. T. Pantev C. (2005). Right hemispheric laterality of human 40 Hz auditory steady-state responses. Cereb. Cortex 15, 2029–2039. 10.1093/cercor/bhi078 15772375

[B91] Ross B. Schneider B. Snyder J. S. Alain C. (2010). Biological markers of auditory gap detection in young, middle-aged, and older adults. PLoS ONE 5:e0010101. 10.1371/journal.pone.0010101 20404929 PMC2852420

[B92] Ross B. Tremblay K. L. Alain C. (2020). Simultaneous EEG and MEG recordings reveal vocal pitch elicited cortical gamma oscillations in young and older adults. Neuroimage 204:116253. 10.1016/j.neuroimage.2019.116253 31600592

[B93] Rueda-Delgado L. M. Solesio-Jofre E. Mantini D. Dupont P. Daffertshofer A. Swinnen S. P. (2017). Coordinative task difficulty and behavioural errors are associated with increased long-range beta band synchronization. Neuroimage 146, 883–893. 10.1016/j.neuroimage.2016.10.030 27771348

[B94] Sawilowsky S. S. (2009). Very large and huge effect sizes. J. Mod. Appl. Stat. Methods 8, 597–599. 10.22237/jmasm/1257035100

[B95] Shalom D. B. Poeppel D. (2008). Functional anatomic models of language: assembling the pieces. Neuroscientist 14, 119–127. 10.1177/1073858407305726 17911215

[B96] Shannon R. V. Zeng F. Kamath V. Wygonski J. Ekelid M. (1995). Speech recognition with primarily temporal cues. Science 270, 303–304. 10.1126/science.270.5234.303 7569981

[B97] Steinmann I. Gutschalk A. (2011). Potential fMRI correlates of 40-Hz phase locking in primary auditory cortex, thalamus and midbrain. Neuroimage 54, 495–504. 10.1016/j.neuroimage.2010.07.064 20688174

[B98] Stone M. A. Füllgrabe C. Moore B. C. (2010). Relative contribution to speech intelligibility of different envelope modulation rates within the speech dynamic range. J. Acoust. Soc. Am. 128, 2127–2137. 10.1121/1.3479546 20968383

[B99] Sullivan G. M. Feinn R. (2012). Using effect size—or why the P value is not enough . J. Grad. Med. Educ. 4, 279–282. 10.4300/JGME-D-12-00156.1 23997866 PMC3444174

[B100] Tadel F. Baillet S. Mosher J. C. Pantazis D. Leahy R. M. (2011). Brainstorm: a user-friendly application for MEG/EEG analysis. Comput. Intell. Neurosci. 2011, 1–13. 10.1155/2011/879716 21584256 PMC3090754

[B101] Tadel F. Bock E. Niso G. Mosher J. C. Cousineau M. Pantazis D.. (2019). MEG/EEG group analysis with brainstorm. Front. Neurosci. 13:76. 10.3389/fnins.2019.00076 30804744 PMC6378958

[B102] Tlumak A. I. Durrant J. D. Delgado R. E. (2015). The effect of advancing age on auditory middle- and long-latency evoked potentials using a steady-state-response approach. Am. J. Audiol. 24, 494–507. 10.1044/2015_AJA-15-0036 26650518

[B103] Vanvooren S. Poelmans H. Hofmann M. Ghesquière P. Wouters J. (2014). Hemispheric asymmetry in auditory processing of speech envelope modulations in prereading children. J. Neurosci. 34, 1523–1529. 10.1523/JNEUROSCI.3209-13.2014 24453339 PMC6705306

[B104] Walton J. P. Frisina R. D. O'Neill W. E. (1998). Age-related alteration in processing of temporal sound features in the auditory midbrain of the CBA mouse. J. Neurosci. 18, 2764–2776. 10.1523/JNEUROSCI.18-07-02764.1998 9502833 PMC6793092

[B105] Walton J. P. Simon H. Frisina R. D. Giraudet P. (2002). Age-related alterations in the neural coding of envelope periodicities. J. Neurophysiol. 88, 565–578. 10.1152/jn.2002.88.2.565 12163510

