# Supplementary material for: Neural Generators Underlying Temporal Envelope Processing Show Altered Responses and Hemispheric Asymmetry Across Age
Source: Front Aging Neurosci. 2020 Dec 4;12:596551. doi: 10.3389/fnagi.2020.596551 (PMC7746817; doi:10.3389/fnagi.2020.596551)
Supplement: Supplementary file 1 [file Data_Sheet_1.pdf]

Supplementary Material

Neural generators underlying auditory temporal processing show altered responses and hemispheric asymmetry across age

Ehsan Darestani Farahani[1*], Jan Wouters[1], Astrid van Wieringen[1]

[1]Research Group Experimental ORL, Department of Neurosciences, KU Leuven, Leuven, Belgium

1. Supplementary Figures

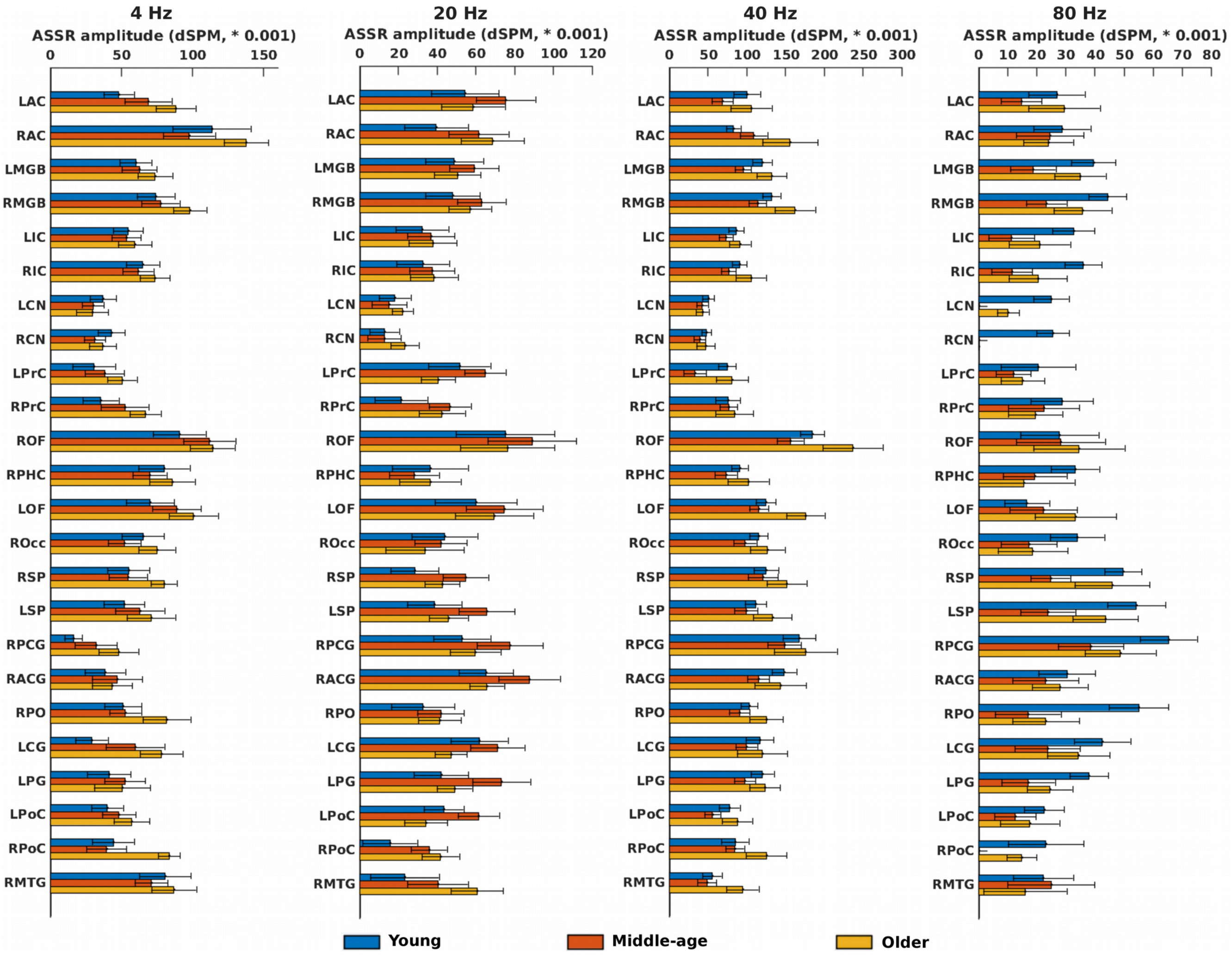

Supplementary Figure 1. ASSR amplitudes of different neural sources (anatomical labels in Table 1) across age (indicated by different colors) in response to 4, 20, 40, and 80 Hz AM stimuli presented to the **left ear**. The magnitudes were expressed in milli-dSPM (i.e., one thousandth of a dSPM). The error-bars illustrate the standard deviations estimated using the Jackknife method.

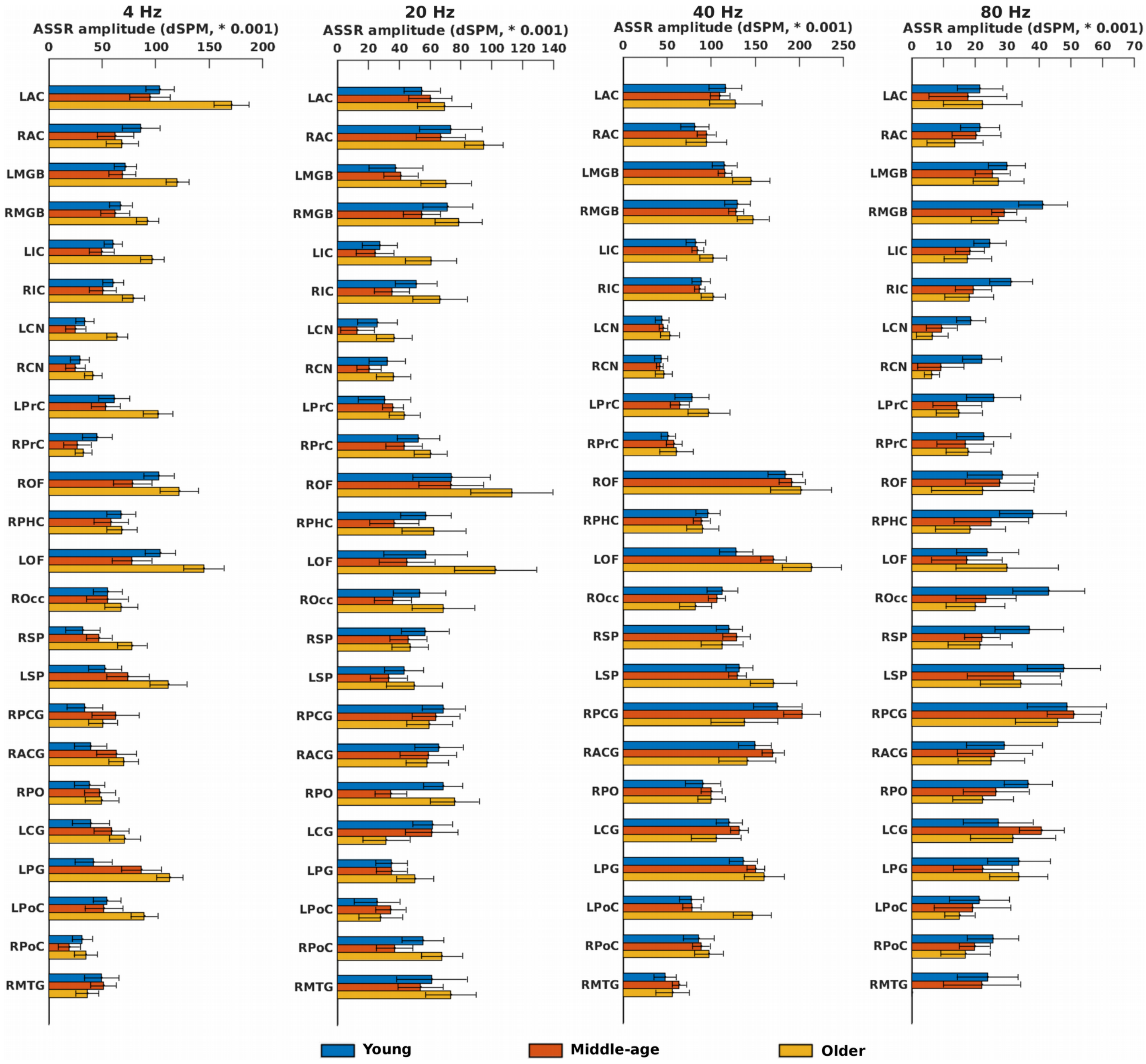

Supplementary Figure 2. ASSR amplitudes of different neural sources (anatomical labels in Table 1) across age (indicated by different colors) in response to 4, 20, 40, and 80 Hz AM stimuli presented to the **right ear**. The magnitudes were expressed in milli-dSPM (i.e., one thousandth of a dSPM). The error-bars illustrate the standard deviations estimated using the Jackknife method.

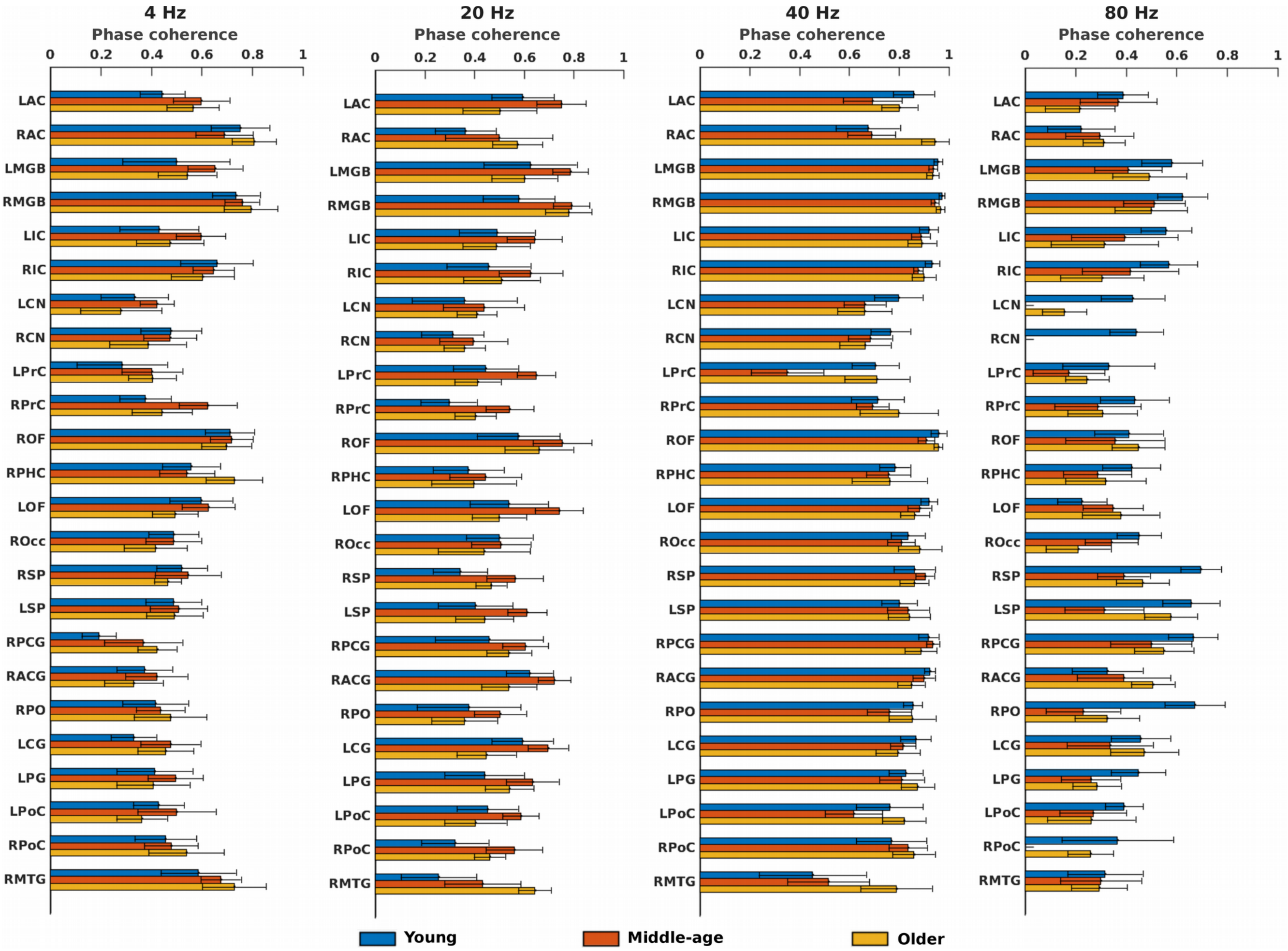

Supplementary Figure 3. Phase coherences of different neural sources (anatomical labels in Table 1) across age (indicated by different colors) in response to 4, 20, 40, and 80 Hz AM stimuli presented to the **left ear**. The error-bars illustrate the standard deviations estimated using the Jackknife method.

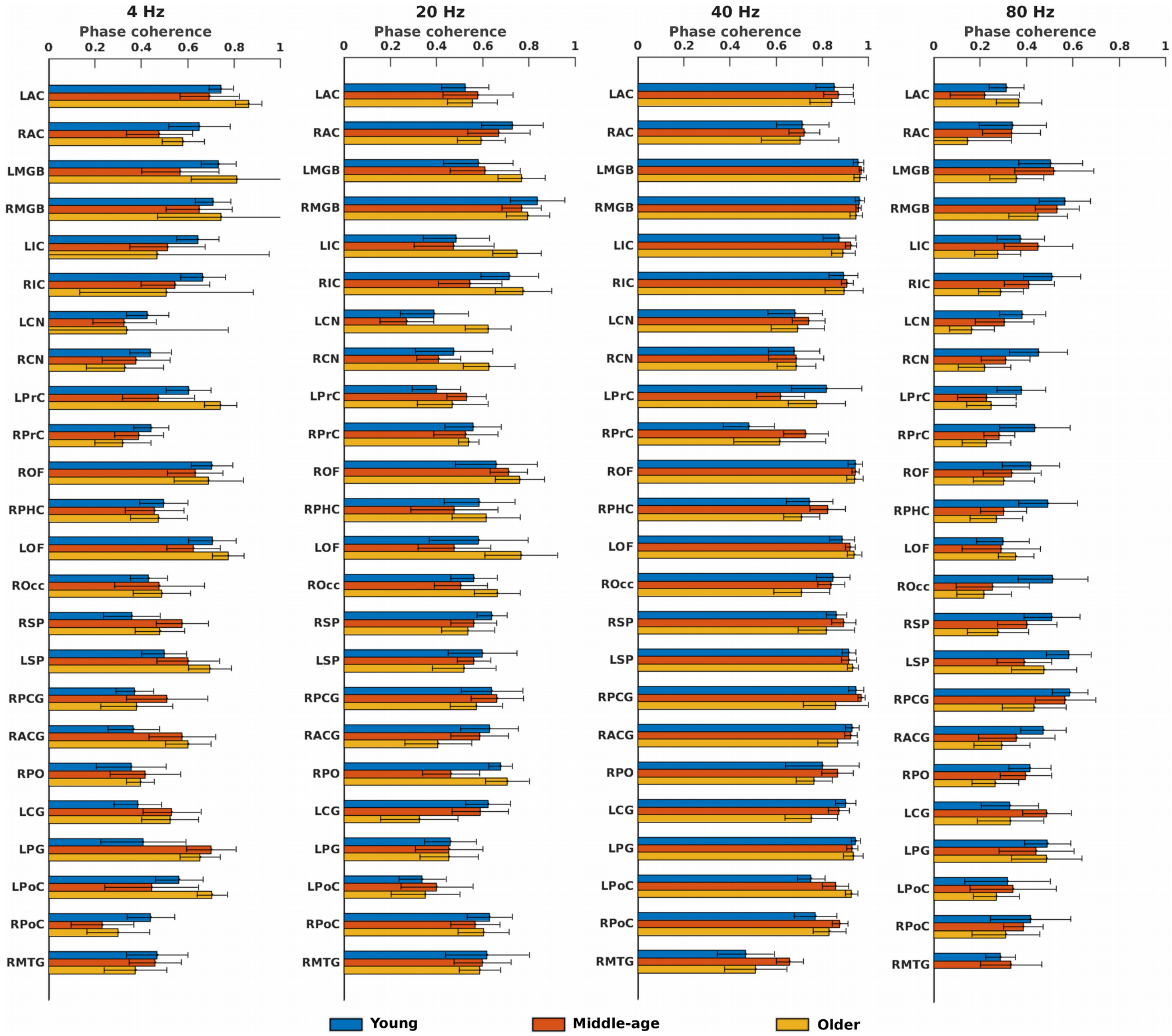

Supplementary Figure 4. Phase coherences of different neural sources (anatomical labels in Table 1) across age (indicated by different colors) in response to 4, 20, 40, and 80 Hz AM stimuli presented to the **right ear**. The error-bars illustrate the standard deviations estimated using the Jackknife method.

2. Supplementary Tabels

Supplementary Table 1. The results of post-hoc comparisons of ASSR amplitudes across age in different neural sources (anatomical labels in Table 1). The results were reported for different pairs of age cohorts (Y: young, M: middle-age, O: older) and different stimulation conditions (4, 20, 40, and 80 Hz AM stimuli presented to the left (L) or right (R) ear). N.S. indicates a non-significant differences.

		4 Hz		20 Hz		40 Hz		80 Hz	
		L	R	L	R	L	R	L	R
LAC	Y, M	t(34)=-4.45 p<.001	N.S.	t(34)=-3.82 p<.001	N.S.	t(31)=5.83 p<.001	N.S.	t(33)=4.17 p<.001	N.S.
	Y, O	t(31)=-9.28 p<.001	t(32)=-13.09 p<.001	N.S.	t(32)=-2.87 p=.007	N.S.	N.S.	N.S.	N.S.
	M, O	t(31)=-3.55 p=.001	t(32)=-12.48 p<.001	t(30)=2.97 p=.005	N.S.	t(28)=-4.86 p<.001	t(31)=-2.37 p=.02	t(28)=-4.06 p<.001	N.S.
RAC	Y, M	N.S.	t(34)=4.11 p<.001	t(34)=-4.12 p<.001	N.S.	t(31)=-5.28 p<.001	t(34)=-2.86 p=.007	N.S.	N.S.
	Y, O	t(31)=-3.00 p=.005	t(32)=3.09 p=.004	t(30)=-4.97 p<.001	t(32)=-3.60 p=.001	t(29)=-8.11 p<.001	N.S.	N.S.	t(31)=2.99 p=.005
	M, O	t(31)=-6.64 p<.001	N.S.	N.S.	t(32)=-5.59 p<.001	t(28)=-4.63 p<.001	N.S.	N.S.	t(31)=2.36 p=.024
LMGB	Y, M	N.S.	N.S.	N.S.	N.S.	t(31)=6.18 p<.001	N.S.	t(33)=7.97 p<.001	t(33)=2.40 p=.022
	Y, O	t(31)=-3.34 p=.002	t(32)=-13.30 p<.001	N.S.	t(32)=-5.62 p<.001	N.S.	t(31)=-4.94 p<.001	N.S.	N.S.
	M, O	t(31)=-2.57 p=.015	t(32)=-12.57 p<.001	N.S.	t(32)=-6.21 p<.001	t(28)=-6.64 p<.001	t(31)=-5.51 p<.001	t(28)=-5.28 p<.001	N.S.
RMGB	Y, M	N.S.	N.S.	t(34)=-3.32 p=.002	t(34)=3.52 p=.001	t(31)=4.45 p<.001	N.S.	t(33)=9.28 p<.001	t(33)=5.94 p<.001
	Y, O	t(31)=-5.46 p<.001	t(32)=-6.81 p<.001	N.S.	N.S.	t(29)=-4.31 p<.001	t(31)=-3.13 p=.003	t(29)=2.90 p=.007	t(31)=4.89 p<.001
	M, O	t(31)=-4.65 p<.001	t(32)=-7.25 p<.001	N.S.	t(32)=-5.05 p<.001	t(28)=-6.69 p<.001	t(31)=-4.03 p<.001	t(28)=-4.05 p<.001	N.S.
LIC	Y, M	N.S.	t(34)=3.09 p=.003	N.S.	N.S.	t(31)=4.15 p<.001	N.S.	t(33)=8.38 p<.001	t(33)=3.78 p<.001
	Y, O	N.S.	t(32)=-10.90 p<.001	N.S.	t(32)=-6.87 p<.001	N.S.	t(31)=-4.32 p<.001	t(29)=3.69 p<.001	t(31)=3.14 p=.003
	M, O	N.S.	t(32)=-12.04 p<.001	N.S.	t(32)=-7.30 p<.001	t(28)=-4.39 p<.001	t(31)=-4.45 p<.001	t(28)=-2.89 p=.007	N.S.
RIC	Y, M	N.S.	t(34)=2.54 p=.015	N.S.	t(34)=3.77 p<.001	t(31)=4.31 p<.001	N.S.	t(33)=10.74 p<.001	t(33)=5.63 p<.001
	Y, O	N.S.	t(32)=-5.47 p<.001	N.S.	t(32)=-2.90 p=.006	t(29)=-2.75 p=.010	t(31)=-3.27 p=.002	t(29)=5.26 p<.001	t(31)=5.24 p<.001
	M, O	t(31)=-3.13 p=.003	t(32)=-7.16 p<.001	N.S.	t(32)=-6.22 p<.001	t(28)=-5.24 p<.001	t(31)=-4.32 p<.001	t(28)=-2.87 p=.007	N.S.

LCN	Y, M	t(34)=2.42 p=.020	t(34)=2.87 p=.006	N.S.	t(34)=3.28 p=.002	t(31)=3.03 p=.004	N.S.	N.S.	t(33)=5.77 p<.001
	Y, O	N.S.	t(32)=-9.57 p<.001	N.S.	t(32)=-2.57 p=.014	t(29)=2.61 p=.014	t(31)=-2.85 p=.007	t(29)=7.40 p<.001	t(31)=7.23 p<.001
	M, O	N.S.	t(32)=-11.80 p<.001	t(30)=-2.50 p=.017	t(32)=-6.17 p<.001	N.S.	t(31)=-2.73 p=.010	N.S.	N.S.
RCN	Y, M	t(34)=4.09 p<.001	N.S.	N.S.	t(34)=3.51 p=.001	t(31)=3.52 p=.001	N.S.	N.S.	t(33)=5.64 p<.001
	Y, O	N.S.	t(32)=-4.14 p<.001	t(30)=-3.95 p<.001	N.S.	N.S.	N.S.	N.S.	t(31)=9.38 p<.001
	M, O	N.S.	t(32)=-5.49 p<.001	t(30)=-3.72 p<.001	t(32)=-4.82 p<.001	N.S.	N.S.	N.S.	N.S.

Supplementary Table 2. The results of post-hoc comparisons of phase coherences across age in different neural sources (anatomical labels in Table 1). The results were reported for different pairs of age cohorts (Y: young, M: middle-age, O: older) and different stimulation conditions (4, 20, 40, and 80 Hz AM stimuli presented to the left (L) or right (R) ear). N.S. indicates a non-significant differences.

		4 Hz		20 Hz		40 Hz		80 Hz	
		L	R	L	R	L	R	L	R
LAC	Y, M	t(34)=-4.61 p<.001	N.S.	t(34)=-4.09 p<.001	N.S.	t(31)=4.68 p<.001	N.S.	N.S.	t(33)=2.31 p=.026
	Y, O	t(31)=-3.64 p<.001	t(32)=-6.35 p<.001	N.S.	N.S.	N.S.	N.S.	t(29)=3.97 p<.001	N.S.
	M, O	N.S.	t(32)=-4.88 p<.001	t(30)=5.62 p<.001	N.S.	t(28)=-2.96 p=.006	N.S.	t(28)=2.77 p=.009	t(31)=-3.33 p=.002
RAC	Y, M	N.S.	t(34)=3.76 p<.001	N.S.	N.S.	N.S.	N.S.	N.S.	N.S.
	Y, O	N.S.	N.S.	t(30)=-5.20 p<.001	t(32)=3.27 p=.002	t(29)=-7.22 p<.001	N.S.	N.S.	t(31)=3.26 p=.002
	M, O	t(31)=-3.28 p=.002	N.S.	N.S.	N.S.	t(28)=-8.70 p<.001	N.S.	N.S.	t(31)=3.42 p=.001
LMG B	Y, M	t(34)=-2.73 p=.009	t(34)=3.82 p<.001	t(34)=-3.40	N.S.	t(31)=3.13 p=.003	N.S.	t(33)=4.01 p<.001	N.S.
	Y, O	N.S.	N.S.	N.S.	t(32)=-4.19 p<.001	t(29)=2.70 p=.011	N.S.	N.S.	t(31)=3.32 p=.002
	M, O	t(31)=2.82 p=.008	t(32)=-3.89 p<.001	t(30)=5.08 p<.001	t(32)=-3.50 p=.001	N.S.	N.S.	N.S.	t(31)=3.19 p=.003
RMG B	Y, M	N.S.	N.S.	t(34)=-5.50 p<.001	N.S.	t(31)=5.72 p<.001	N.S.	t(33)=2.96 p=.005	N.S.
	Y, O	N.S.	N.S.	t(30)=-4.48 p<.001	N.S.	N.S.	N.S.	t(29)=2.84 p=.008	t(31)=2.76 p=.009
	M, O	N.S.	N.S.	N.S.	N.S.	t(28)=-3.47 p=.001	N.S.	N.S.	N.S.

LIC	Y, M	t(34)=-3.78 p<.001	t(34)=2.98 p=.005	t(34)=-3.39 p=.001	N.S.	N.S.	t(34)=-2.86 p=.007	t(33)=2.97 p=.005	N.S.
	Y, O	N.S.	N.S.	N.S.	t(32)=-6.06 p<.001	N.S.	N.S.	t(29)=4.27 p<.001	t(31)=2.81 p=.008
	M, O	t(31)=2.98 p=.005	N.S.	t(30)=3.52 p=.001	t(32)=-5.48 p<.001	N.S.	t(31)=2.49 p=.018	N.S.	t(31)=4.00 p<.001
RIC	Y, M	N.S.	t(34)=2.83 p=.007	t(34)=-3.39 p=.001	t(34)=3.91 p<.001	t(31)=6.38 p<.001	N.S.	t(33)=2.89 p=.006	t(33)=2.55 p=.015
	Y, O	N.S.	N.S.	N.S.	N.S.	N.S.	N.S.	t(29)=5.24 p<.001	t(31)=5.72 p<.001
	M, O	N.S.	N.S.	N.S.	t(32)=-5.17 p<.001	N.S.	N.S.	N.S.	t(31)=3.44 p=.001
LCN	Y, M	t(34)=-2.50	t(34)=2.58 p=.014	N.S.	t(34)=2.68 p=.01	t(31)=4.29 p<.001	N.S.	N.S.	N.S.
	Y, O	N.S.	N.S.	N.S.	t(32)=-5.37 p<.001	t(29)=3.68 p<.001	N.S.	t(29)=6.47 p<.001	N.S.
	M, O	t(31)=3.41 p=.001	N.S.	N.S.	t(32)=-9.42 p<.001	N.S.	N.S.	N.S.	t(31)=3.49 p=.001
RCN	Y, M	N.S.	N.S.	N.S.	N.S.	t(31)=2.78 p=.008	N.S.	N.S.	t(33)=3.62 p<.001
	Y, O	N.S.	N.S.	N.S.	t(32)=-3.07 p=.004	t(29)=3.13 p=.003	N.S.	N.S.	t(31)=5.47 p<.001
	M, O	N.S.	N.S.	N.S.	t(32)=-6.14 p<.001	N.S.	N.S.	N.S.	t(31)=2.39 p=.023

Supplementary Table 3. The result of the tests for hemispheric lateralization of the neural source in the auditory cortex (AC) in different age cohorts (Y: young, M: middle-age, O: older) and different stimulation conditions (4, 20, 40, and 80 Hz AM stimuli presented to the left (L) or right (R) ear). N.S. indicates a non-significant differences.

		**4 Hz**		**20 Hz**		**40 Hz**		**80 Hz**	
		**L**	**R**	**L**	**R**	**L**	**R**	**L**	**R**
**AC**	**Y**	t(17)=13.45 p < 0.001	t(17)=-3.82 p=0.001	t(17)=-4.27 p < 0.001	t(17)=4.18 p < 0.001	t(16)=-4.09 p < 0.001	t(17)=-7.23 p < 0.001	N.S.	N.S.
	**M**	t(17)=5.87 p < 0.001	t(17)=-11.83 p<0.001	t(17)=-3.72 p < 0.01	N.S.	t(15)=12.13 p < 0.001	t(17)=-6.75 p < 0.001	t(15)=3.51 p < 0.01	N.S.
	**O**	t(14)=10.23 p < 0.001	t(15)=-20.62 p < 0.001	N.S.	t(15)=5.37 p < 0.001	t(13)=4.55 p < 0.001	t(14)=-7.46 p < 0.001	N.S.	t(14)=-3.86 p < 0.01

Supplementary Table 4. The result of the tests for hemispheric lateralization of the subcortical sources (the medial geniculate body (MGB), the inferior colliculus (IC), the cochlear nucleus (CN)) in different age cohorts (Y: young, M: middle-age, O: older) in response to 80 Hz AM stimuli presented to the left (L) or right (R) ear. N.S. indicates a non-significant differences.

		**L**	**R**
**MGB**	**Y**	$t(17)=4.13$ $p<0.001$	$t(16)=16.93$ $p<0.001$
	**M**	$t(16)=2.88$ $p<0.05$	$t(17)=3.66$ $p<0.01$
	**O**	N.S.	N.S.
**IC**	**Y**	$t(17)=4.39$ $p<0.001$	$t(16)=11.04$ $p<0.001$
	**M**	N.S.	N.S.
	**O**	N.S.	N.S.
**CN**	**Y**	N.S.	$t(16)=5.58$ $p<0.001$
	**M**	-	N.S.
	**O**	-	N.S.
